# Pore-in-Pore Engineering in a Covalent Organic Framework Membrane for Gas Separation

**DOI:** 10.1021/acsnano.2c12774

**Published:** 2023-04-07

**Authors:** Hongwei Fan, Haoran Wang, Manhua Peng, Hong Meng, Alexander Mundstock, Alexander Knebel, Jürgen Caro

**Affiliations:** † College of Chemical Engineering, Beijing University of Chemical Technology, Beijing 100029, PR China; ‡ Institute of Physical Chemistry and Electrochemistry, Leibniz Universität Hannover, Callinstraße 3A, 30167 Hannover, Germany; § Key Laboratory of Power Station Energy Transfer Conversion and System, Ministry of Education, School of Energy Power and Mechanical Engineering, North China Electric Power University, Beijing 102206, PR China; # Otto Schott Institute of Materials Research, Friedrich Schiller University Jena, Fraunhoferstraße 6, 07743 Jena, Germany

**Keywords:** molecular-separation membrane, covalent organic framework, pore-in-pore engineering, gas separation, *in situ* interfacial polymerization

## Abstract

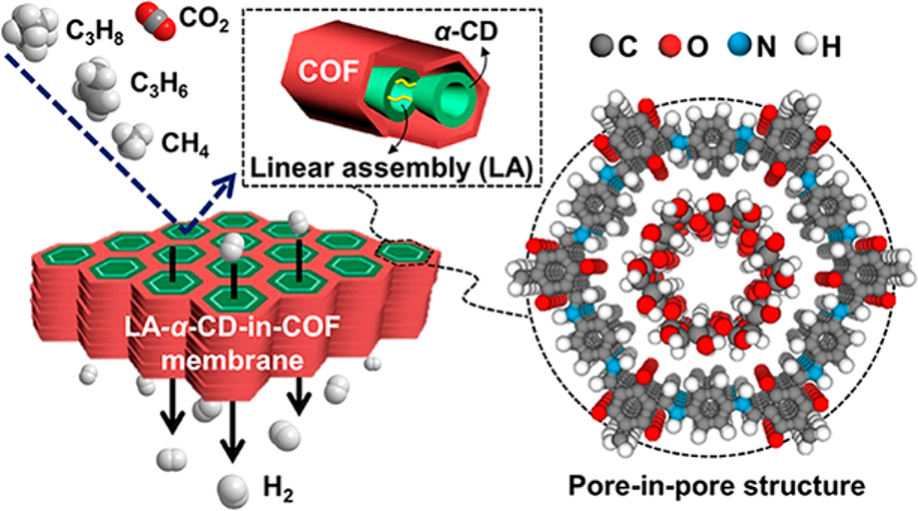

Covalent organic framework (COF) membranes have emerged as a promising candidate for energy-efficient separations, but the angstrom-precision control of the channel size in the subnanometer region remains a challenge that has so far restricted their potential for gas separation. Herein, we report an ultramicropore-in-nanopore concept of engineering matreshka-like pore-channels inside a COF membrane. In this concept, α-cyclodextrin (α-CD) is in situ encapsulated during the interfacial polymerization which presumably results in a linear assembly (LA) of α-CDs in the 1D nanochannels of COF. The LA-α-CD-in-TpPa-1 membrane shows a high H_2_ permeance (∼3000 GPU) together with an enhanced selectivity (>30) of H_2_ over CO_2_ and CH_4_ due to the formation of fast and selective H_2_-transport pathways. The overall performance for H_2_/CO_2_ and H_2_/CH_4_ separation transcends the Robeson upper bounds and ranks among the most powerful H_2_-selective membranes. The versatility of this strategy is demonstrated by synthesizing different types of LA-α-CD-in-COF membranes.

Covalent organic frameworks (COFs) are an emerging class of porous crystalline polymers connected by organic building units through covalent bonds into highly ordered and periodic network structures.^1−3^ These materials have gained tremendous attention because of their potential applications in diverse fields such as gas adsorption for separation and storage, catalysis, energy storage, optoelectronics, and many more.^4−7^ Given by the versatile architectures, tunable functionalities, and well-organized pore system as well as good thermal and chemical stability, the COFs, especially the Schiff base-related 2D COF family, hold great potential for energy-efficient membrane-based molecular/ion separations in the chemical industry.^8−13^ For this purpose, the development of COF membranes has attracted extensive interest in the last five years and is booming right now.^14−18^ A variety of self-supporting or supported high-quality COF membranes were developed with a fascinating performance in liquid-phase separation processes^19^ such as desalination,^20,21^ dye wastewater purification,^22−24^ and organic solvent nanofiltration.^25−27^ However, progress on COF-based membranes in selective gas separation is lagging, mainly due to the intrinsic nanometer-sized pores of the COF family (typically 0.6–10 nm) which are much larger than the kinetic diameter of ordinary gas molecules (0.25–0.5 nm).^28−31^ Based on the topology diagrams and pore-wall surface engineering,^32−34^ it is difficult to design the COFs with an aperture size in the gas molecular-selective region. Approaches including staggered stacking,^35−39^ oriented growth,^40^ and hybridization with other microporous nanomaterials^41,42^ have been explored to reduce the effective pore size of COF membranes toward the ultramicroporous range, mainly aiming at improving the molecular sieving mechanism. Even so, to realize a tuning of the channel size with Angstrom-precision still remains a great challenge, and therefore, the COF membrane performance for gas separation is often limited.

Host–guest inclusion complexes are an interesting configuration in which a small “guest” molecule is included within the interior of a porous macromolecular “host” compound.^43,44^ Such nanocomposite materials often possess synergistic functionalities, providing significantly enhanced properties in comparison to those of their individual counterparts.^45−48^ COFs having a 1D pore channel or a 3D “cage-like” pore system are ideal host matrixes for accommodating nanoentities such as metal nanoparticles, quantum dots, organic and metal–organic molecules, biomacromolecules, metal–organic polyhedra, porous organic cages, and metal–organic frameworks (MOFs).^49−52^ The encapsulation of these nanoentities in COFs has led to the development of functional materials for adsorption and separation, sensing, heterogeneous catalysis, energy harvesting, and molecular release systems.^53−55^ One representative group is the β-CD (β-cyclodextrin)-decorated COF nanochannels which enable an enantioselective transport of amino acids.^56^ Another example is the COF nanocomposite membrane which contains a unit cell-sized MOF, exhibiting a more precise molecular sieving for selective H_2_ separation.^57^ Thus, the construction of hybrid COFs as host–guest complex offers a rich playground to design gas separation membrane materials.

Inspired by the host–guest hybrid nanocomposites, in this study, we present a pore-in-pore engineering concept of packing linear cyclodextrin (CD) polymers into the 1D nanochannels of 2D COF to fabricate hierarchical-structured COF membranes. These membranes have matreshka-like ultramicropore-in-nanopore channels consisting of fast and H_2_-preferential transport pathways, which are expected to exhibit an excellent H_2_ permeance and high selectivity in mixed-gas permeation. The pore-in-pore strategy provides another way to tune the pore environment of COFs for advanced molecular-separation membranes.

## Results and Discussion

### Preparation of LA-α-CD-in-COF Membrane

**Scheme 1:**
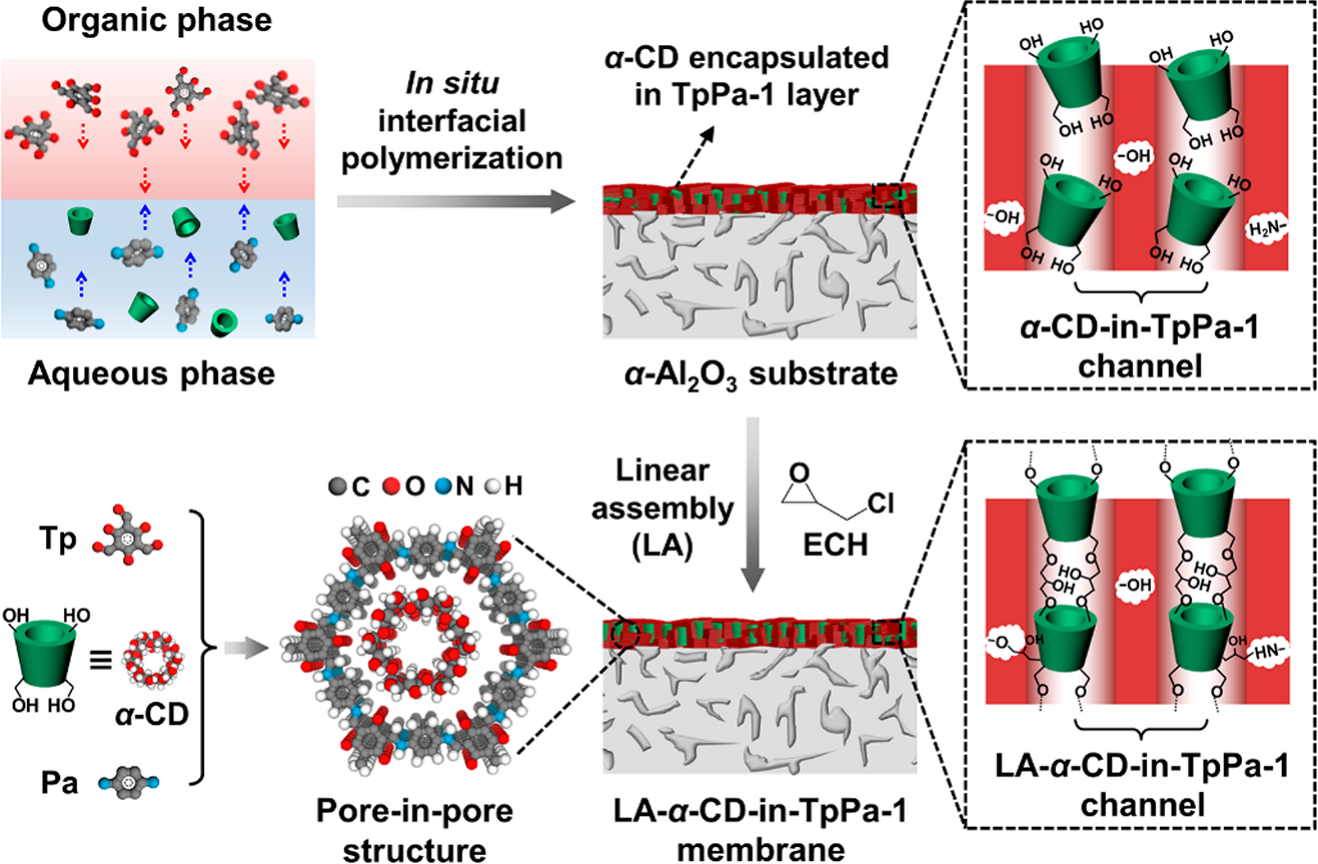
LA-α-CD-in-TpPa-1 Membrane Synthesis and Schematic of Pore-in-Pore Structure

To realize the pore-in-pore concept, α-cyclodextrin (α-CD) is selected as the building block for ultramicropore channels inside the 2D ketoenamine-linked 1,3,5-triformylphloroglucinol (Tp) *p*-phenylenediamine (Pa-1) (TpPa-1) membrane. The α-CD is a cylinder-shaped macromolecule, composed of six glucose units, which not only has a tiny cavity diameter (0.47–0.53 nm) but also a desirable molecular dimension of about 1.37 nm (Figure S1) smaller than the 1D nanopore channels of the TpPa-1 (∼1.8 nm).^58,59^ Moreover, the α-CD molecules could be assembled into linear polymers through a preprogrammed cross-linking reaction (linear assembly (LA)).^60,61^ The packing of linear α-CD polymers into the TpPa-1 nanochannel would create numerous selective transport pathways for the targeted gas components in the resulting membrane. This strategy is demonstrated via the preparation of the LA-α-CD-in-TpPa-1 membrane (as illustrated in Scheme 1), which was accomplished by a facile two-step procedure. First, α-CD is embedded during the in situ interfacial polymerization to form an α-CD encapsulated TpPa-1 layer onto the amino-modified α-Al_2_O_3_ substrate surface. It is expected that the α-CD molecules are packed inside the ordered 1D column nanochannels accompanied by the TpPa-1 crystal nucleation and growth process. Thereafter, the supported α-CD-in-TpPa-1 layer was immersed into an epichlorohydrin (ECH) alkaline solution to allow the reaction between the encapsulated α-CD molecules with the epoxide ring of ECH, resulting in glyceryl bridges between neighboring α-CD molecules in the confined space of 1D COF channel. It should be noted that also reactions between ECH and nonreacted COF monomers (−OH, −NH_2_) might happen because the COF structure is rather difficult to get 100% perfect during the membrane formation. These potential reactions would immobilize α-CDs onto the COF pore walls and promote the formation of linearly oriented α-CDs inside the COF pores. Finally, the LA-α-CD-in-TpPa-1 membrane was obtained after a rinsing treatment.

### Morphological and Structural Characterization of the LA-α-CD-in-COF Membrane

**Figure 1:**
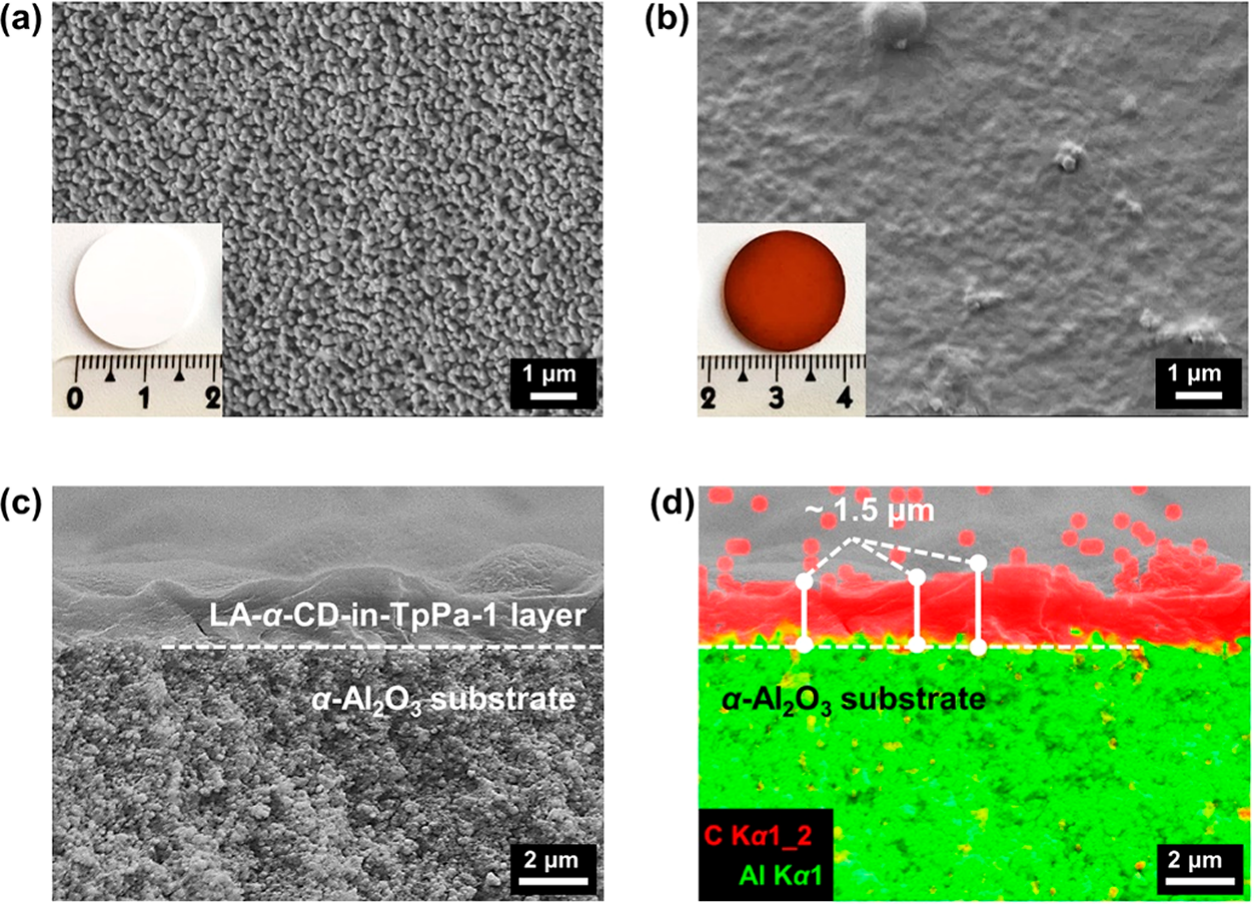
Top-view SEM images of (a) α-Al_2_O_3_ substrate and (b) LA-α-CD-in-TpPa-1 membrane. The inserted digital photographs in the bottom-left corner of (a) and (b) correspond to pure α-Al_2_O_3_ substrate in white and the LA-α-CD-in-TpPa-1 membrane in crimson, respectively. (c) Cross-sectional SEM images of LA-α-CD-in-TpPa-1 membrane. (d) EDXS mapping with elemental distributions corresponding to (c).

The white α-Al_2_O_3_ disk became crimson after the COF synthesis due to the characteristic color of TpPa-1 (bottom-left corners in Figure 1a,b). The top-view SEM image (Figure 1b) reveals a continuous LA-α-CD-in-TpPa-1 layer without visible cracks or pinholes covering the porous α-Al_2_O_3_ disk surface (Figure 1a). Moreover, according to cross-sectional SEM images (Figure S2 and Figure 1c), the layer is about 1.5 μm in thickness grown on the α-Al_2_O_3_ substrate. Energy-dispersive X-ray spectroscopy (EDXS) mapping (Figure 1d, corresponding to Figure 1c) shows that there is a sharp transition between the LA-α-CD-in-TpPa-1 layer (C signals, red) and the α-Al_2_O_3_ substrate (Al signals, green), indicating no detectable LA-α-CD-in-TpPa-1 formed in the bulk ceramic substrate.

**Figure 2:**
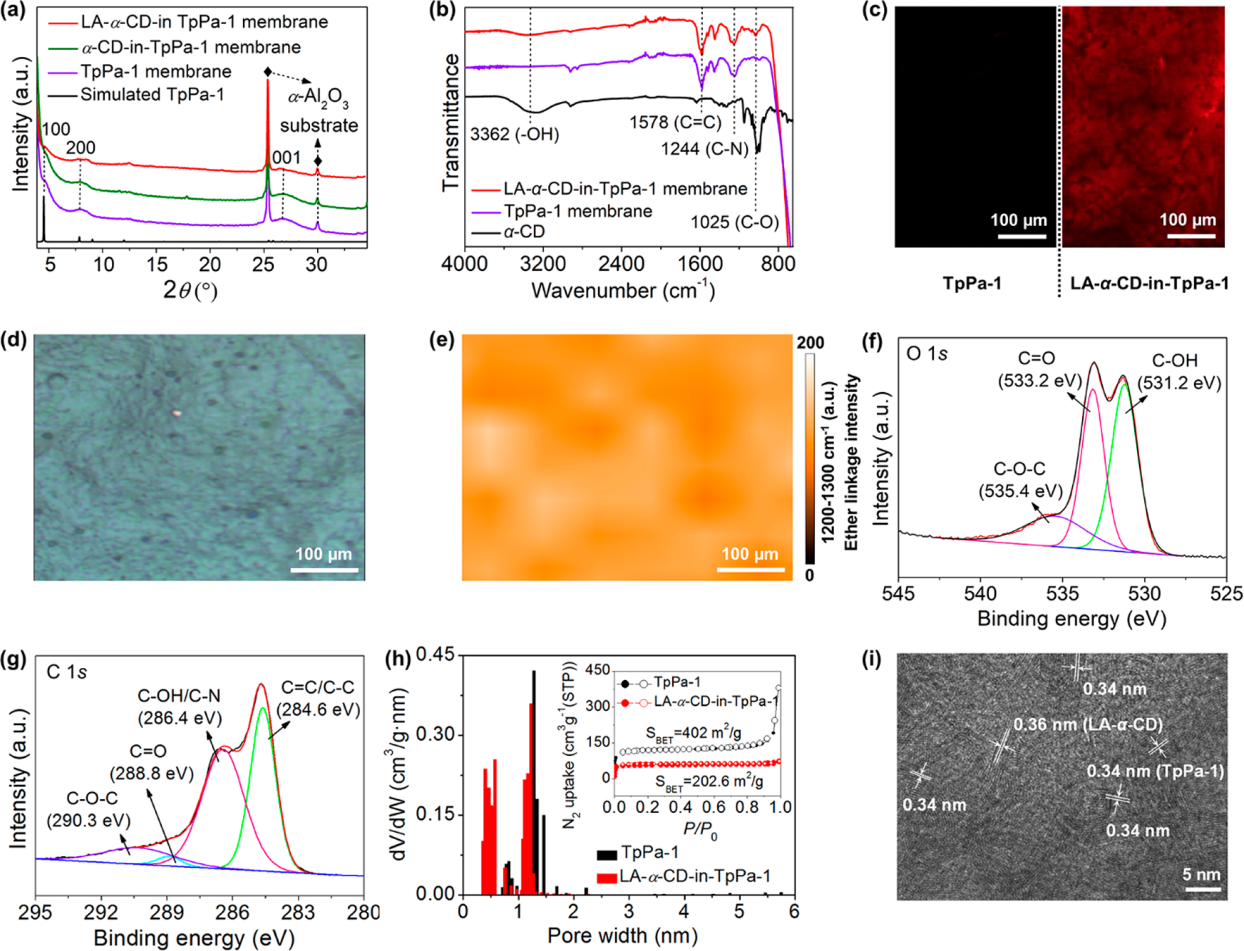
(a) XRD patterns. (b) ATR-FTIR spectra. (c) Surface fluorescence images of TpPa-1 membrane (left) and LA-α-CD-in-TpPa-1 membrane (right) (fluorescein:rhodamine B; excitation wavelength: 546–560 nm). (d) Surface optical microscopy image of the LA-α-CD-in-TpPa-1 membrane for Raman mapping. (e) Raman mapping ranging from 1200 to 1300 cm^–1^ over the area in (d). High-resolution XPS spectra of deconvoluted (f) O1*s* and (g) in the LA-α-CD-in-TpPa-1. (h) Pore-size distribution of TpPa-1 and LA-α-CD-in-TpPa-1 with inserted nitrogen adsorption–desorption isotherms measured at 77 K (adsorption, closed; desorption, open symbols). (i) High-resolution TEM image of LA-α-CD-in-TpPa-1 layer.

As shown in Figure 2a, the supported TpPa-1 layer (TpPa-1 membrane), the supported α-CD-in-TpPa-1 layer (α-CD-in-TpPa-1 membrane), and the supported LA-α-CD-in-TpPa-1 layer (LA-α-CD-in-TpPa-1 membrane) have similar X-ray diffraction (XRD) peaks at ∼4.7°, ∼8.1°, and ∼27° (2θ), corresponding to the (100), (200), and (001) lattice plane reflections of TpPa-1, respectively. These findings prove successful synthesis of TpPa-1 on the substrate and that no structural damage occurred during the α-CD packing and cross-linking inside the 1D COF pore. The XRD diffraction signals of the COF membrane layer are not as strong as those of the COF powders (Figure S1) and the prepared self-supporting COF layer (Figure S3), mainly due to the thinner thickness of about 1.5 μm and much smaller amount compared to the Al_2_O_3_ disk which led to the dominating diffraction signals of the Al_2_O_3_ corundum substrate.^24,62,63^ There are no obvious characteristic diffraction signals of α-CD (Figure S1) in the LA-α-CD-in-TpPa-1 membrane, probably due to the low content (about ∼11.8 vol%) and molecular-level distribution of α-CD molecules assembled inside the 1D nanochannels of TpPa-1. Otherwise, the diffraction peaks of the α-CD aggregates with the cage-type structure^64,65^ should have been detected as we have proven by studying a powder mixture of α-CD and TpPa-1 (Figure S4). Attenuated Total Reflectance-Fourier Transformed InfraRed (ATR-FTIR) spectra (Figure 2b) of the TpPa-1 membrane and LA-α-CD-in-TpPa-1 membrane show strong signals at about 1578 and 1244 cm^–1^, attributed to the characteristic C=C and C–N stretching of the TpPa-1 with a ketoenamine form. The broad adsorption band at about 3362 cm^–1^ is designated to the −OH stretching vibration, and the band at 1025 cm^–1^ is ascribed to the C–O stretching vibration in the LA-α-CD (and/or α-CD). The intensity of these two peaks in the LA-α-CD-in-TpPa-1 membrane is relatively weak because of the low content of LA-α-CD in the TpPa-1 layer. Spatial arrangement of the LA-α-CD in the membrane could be exposed by fluorescence spectroscopy on the basis of the host–guest interaction between LA-α-CD (and/or α-CD) and a fluorescence probe such as rhodamine B. It follows from Figure 2c and Figure S5 that red fluorescence can be clearly observed on the whole surface and cross-section of the LA-α-CD-in-TpPa-1 membrane as compared to the α-CD-free TpPa-1 membrane, evidently indicating the existence of LA-α-CD (and/or α-CD).

Raman and X-ray photoelectron spectroscopy (XPS) were performed to further detect the membrane structure. Surface and cross-sectional Raman mappings (Figure 2e, corresponding to Figure 2d; Figure S6b corresponding to Figure S6a) both display the evenly distributed ether linkages (C–O–C) over the LA-α-CD-in-TpPa-1 membrane as compared to the TpPa-1 powders (Figure S7) and TpPa-1 membrane without α-CD (Figure S8), confirming the uniformity of LA-α-CD (and/or α-CD) incorporated inside the TpPa-1 layer on a macroscopic scales. The full XPS survey spectra (Figure S9a) indicate the presence of nitrogen, carbon, and oxygen in the LA-α-CD-in-TpPa-1 membrane. The high-resolution spectrum of deconvoluted N1*s* (Figure S9b) shows the characteristic energy peaks of TpPa-1 matrix. From the high-resolution spectra of O1*s* (Figure 2f), a binding energy at 535.4 2 eV is assigned to the C–O–C bond of the LA-α-CD (and/or α-CD), and also the relevant characteristic energy peaks could be found in the high-resolution spectra of deconvoluted C1*s* (Figure 2g). Moreover, it can be reasonably deduced that the ultramicropore-in-nanopore structure would be formed if the LA-α-CD (and/or α-CD) was packed inside the 1D nanochannels of TpPa-1, which can be indirectly proved from the changes in specific surface area and pore size distribution. For this purpose, samples of the self-supporting TpPa-1 layer and LA-α-CD-in-TpPa-1 layer were synthesized for the measurement of nitrogen adsorption–desorption isotherms (Figure 2h). The calculated Brunauer–Emmett–Teller (BET) surface area of LA-α-CD-in-TpPa-1 is 203 m^2^·g^–1^, which is much smaller than that of TpPa-1 with 402 m^2^·g^–1^, indicating the partial pore filling with the LA-α-CD (and/or α-CD). It is worth noting that the BET surface areas of both TpPa-1 and LA-α-CD-in-TpPa-1 are comparable to that of the reported TpPa-1 powders and TpPa-1 membranes in the literature.^63,66,67^ However, due to the large amounts of staggered and thus interrupted pore channels, the nitrogen adsorption is affected, and the LA-α-CD-in-TpPa-1 and TpPa-1 layers usually show relatively low BET values compared to powder samples (Figure S10). Notably, the experimental pore size distribution of the LA-α-CD-in-TpPa-1 layer is concentrated in the ultramicroporous region of 0.3–0.5 nm compared with the α-CD-free TpPa-1 (see the inset in Figure 2h), which certifies that the encapsulation of LA-α-CD (and/or α-CD) has indeed generated ultramicropore channels. Besides, the more exciting thing was that the lattice fringes (with a lattice space of 0.36 nm) related to the LA-α-CD were clearly observed from the high-resolution TEM image of the LA-α-CD-in-TpPa-1 layer after being compared with that of LA-α-CD and α-CD (Figure S11). This finding could serve as compelling evidence for the LA of α-CD molecules to be the linear channel-type α-CD structure in the compact TpPa-1 layer. These results collectively demonstrate the successful generation of the ultramicropore-in-nanopore LA-α-CD-in-TpPa-1 membrane with the designed matreshka-like pore-channel structure (Scheme 1).

It is worth mentioning that there are several possible cases of structural arrangement for the LA-α-CD molecules (including some ideal cases, Figure S12a–e). However, inside the confined space of the 1D channels of 2D COF, it could be rationally inferred that the most possible case is the statistically channel-type linear arrangement in which α-CD molecules are connected together and stacked on top of each other (head-to-head or head-to-tail orientation) by cylindrical columns (Figure S12f). Moreover, the α-CD molecules in the linear LA-α-CD polymers may not be strictly aligned. In addition, determining the number of α-CD molecules assembled is indeed interesting but also extremely difficult mainly due to the tiny size and content which is often below the detection limit. We have tried a variety of methods such as high-resolution scanning tunneling microscopy (STM) to figure out the specific shape of LA-α-CD in the membrane, but the results were negative owing to the low image contrast caused by the similar elemental composition to the TpPa-1. Despite this, whatever the length (dimer, trimer, tetramer...), the pore-in-pore structures induced by various channel-type LA-α-CD will be conducive to creating selective transport pathways in the 1D pore channels of COF for the targeted gas molecules such as H_2_ through the membrane.

### Gas-Separation Performance of the LA-α-CD-in-COF Membrane

**Figure 3:**
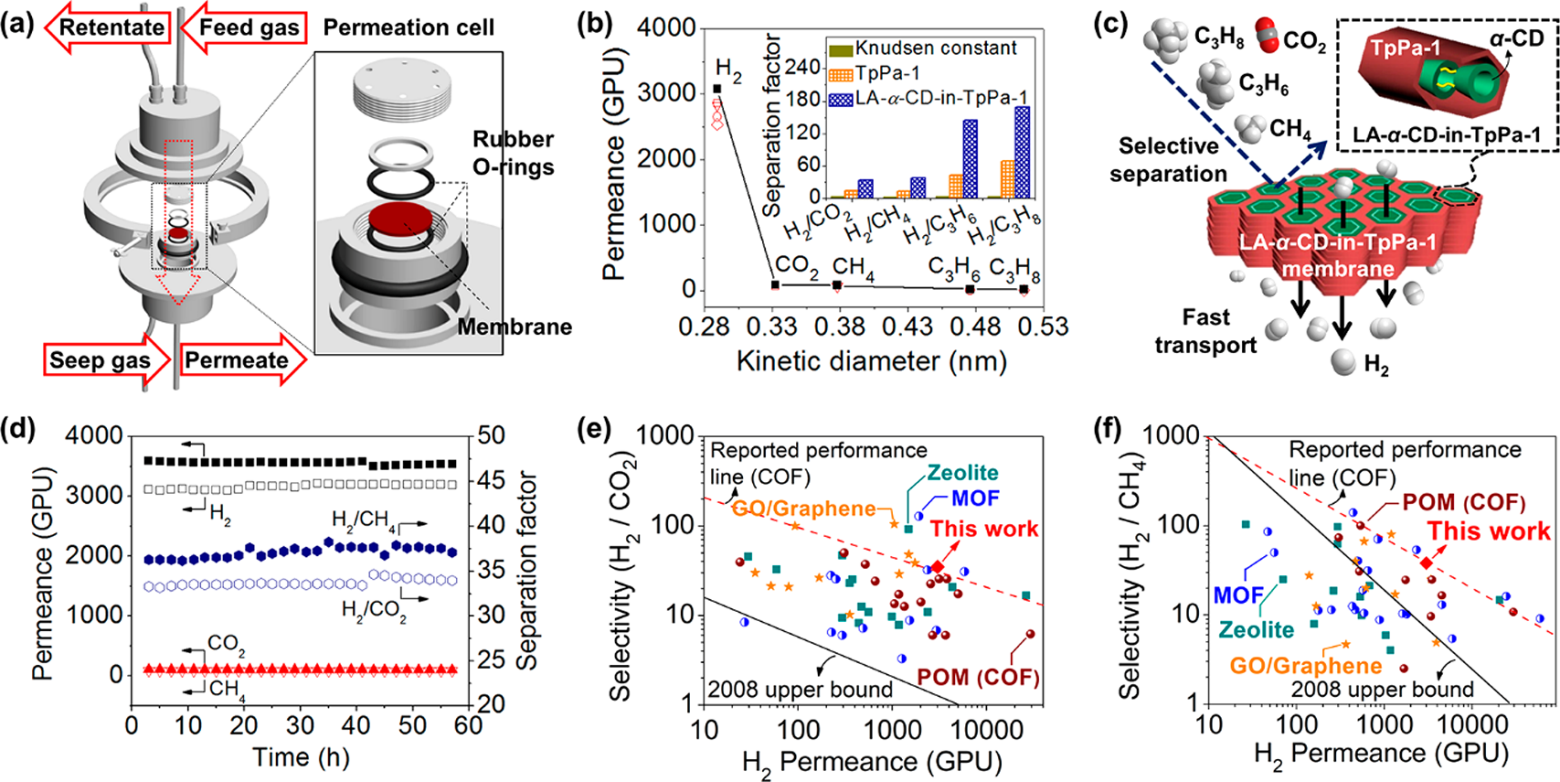
(a) Schematic of membrane module for gas permeation. (b) Permeances of single gases (■) and components of the binary gas mixtures H_2_/CO_2_ (□), H_2_/CH_4_ (▽), H_2_/C_3_H_6_ (○), and H_2_/C_3_H_8_ (◇) on LA-α-CD-in-TpPa-1 membrane (synthesized from an α-CD concentration of 1.4 mg/mL in the precursor solution) as a function of kinetic diameter of permeating molecules at 298 K and 1 bar. The inset shows the mixed-gas separation factor of TpPa-1 membrane and LA-α-CD-in-TpPa-1 membrane. (c) Schematic illustration of gas transport through LA-α-CD-in-TpPa-1 membrane. (d) Long-term tests of LA-α-CD-in-TpPa-1 membrane for equimolar gas mixture of H_2_/CO_2_, and H_2_/CH_4_ at 298 K at 1 bar. Mixed-gas selectivity of (e) H_2_/CO_2_ and (f) H_2_/CH_4_, as a function of H_2_ permeance for LA-α-CD-in-TpPa-1 membrane compared with literature data. Detailed information on the data points is given in Tables S3 and S4.

Gas-separation performance was measured following the Wicke–Kallenbach method (Figure 3a) by placing the membrane into a homemade permeation apparatus (Figure S13). The fluxes of the single gases H_2_, CO_2_, CH_4_, C_3_H_6_, and C_3_H_8_ as well as equimolar (1:1) binary mixtures of H_2_ with CO_2_, CH_4_, C_3_H_6_, and C_3_H_8_ were tested at room temperature (298 K) and 1 bar, respectively. It can be seen that the LA-α-CD-in-TpPa-1 membrane shows a single component H_2_ permeance of 3077.3 GPU, which is much higher than those of the other gases (Figure 3b). The calculated ideal selectivities of H_2_ from CO_2_, CH_4_, C_3_H_6_, and C_3_H_8_ are 36.2, 39.4, 157.8, and 187.1, fairly surpassing the corresponding Knudsen constants (4.7, 2.8, 4.6, and 4.7). This demonstrates the superior H_2_-permselective properties of the LA-α-CD-in-TpPa-1 membrane, which is expected to display a desirable separation performance in mixed-gas permeation. The real selectivities (or separation factors) of the LA-α-CD-in-TpPa-1 membrane for equimolar H_2_/CO_2_, H_2_/CH_4_, H_2_/C_3_H_6_, and C_3_H_8_ gas pairs can reach 34.8, 38.1, 144.6, and 169.2, respectively. A significant improvement in separation selectivity is observed as compared to that of the TpPa-1 membrane without LA-α-CD (Figure S14). Meanwhile, the LA-α-CD-in-TpPa-1 membrane still has a high H_2_ permeance of ∼3000 GPU during the mixed-gas permeation. This result further suggests the formation of an ultramicroporous structure with fast and selective transport channels for H_2_ in the LA-α-CD-in-TpPa-1 membrane (Figure 3c). Moreover, the excellent H_2_ permeance implies a partial filling by LA-α-CD rather than an entire occupancy of the 1D nanochannel of TpPa-1 in view of the low content (∼11.8 vol % estimated based on the C–O–C/C=O atomic (molar) ratio from XPS), thereby leading to only a small mass transfer resistance. We also investigated the effect of the α-CD concentration in the precursor solution on the membrane performance (Figure S15a). As the α-CD concentration increases, the H_2_ permeance decreases gradually. Simultaneously, the separation selectivity of H_2_/CH_4_ went up first and then started to descend when the α-CD concentration reached 2.1 mg/mL. A higher concentration of α-CD makes the COF layer more rigid and brittle, which is prone to have crack defects during the solvent evaporation and drying process (Figure S15b), resulting in a drop of selectivity.

In addition, the LA-α-CD-in-TpPa-1 membrane exhibits a good running stability during a 60 h long continuous gas-permeation measurement (Figure 3d) because the separation performance for equimolar H_2_/CO_2_ and H_2_/CH_4_ mixtures was scarcely deteriorated. Considering practical applications, gas permeation was also conducted at higher temperature to investigate the thermal stability of our membrane. We find a gradual decline of selectivity in H_2_/CO_2_ separation with the increase of testing temperature (Figure S16a), which is due to the stronger activated diffusion of CO_2_ than H_2_ (Figure S16b)_._ Damage to the membrane structure, in terms of a thermal decomposition of TpPa-1 and α-CD, can be excluded from the data. The membrane remains stable at 180 °C, and no performance degradation was observed after a continuous operation over 70 h (Figure S17). In contrast, the α-CD-in-TpPa-1 membrane without LA chain formation through ECH treatment was also prepared and subjected to a long-term gas-permeation study. Both H_2_ permeance and separation selectivity of the H_2_/CO_2_ mixture descend during the testing process (Figure S18). A shifting and joggling of the unbound α-CDs inside the COF nanopores interrupts and blocks the selective gas transport through the α-CDs, which slowly form nonselective diffusion channels in the membrane. This finding also indicates that an LA is critical and necessary to obtain a robust membrane for selective H_2_ separation following our pore-in-pore concept.

Besides temperature, feed pressure is also an important factor influencing the membrane performance. For our LA-α-CD-in-TpPa-1 membrane, the permeances of both H_2_ and CO_2_ increased, and meanwhile, the H_2_/CO_2_ separation factor decreased first from 33 at 1 bar to 17 at 1.2 bar and then leveled off with the further increase of feed pressure (Figure S19). The decrease of separation selectivity is probably related to the flexibility of the channel-type LA-α-CD structure, which might slightly dilate the transport channels formed between the inner wall of COF pore and the exterior wall of LA-α-CD under pressurization, causing the penetration of more CO_2_ molecules. It is noteworthy that the cases such as structural damage and defects could be reasonably ruled out because the LA-α-CD-in-TpPa-1 membrane exhibited an excellent pressure resistance even at 6 bar in a cross-flow nanofiltration test (Figure S20) by using the water and Na_2_SO_4_ aqueous solution as the feed, respectively (Figures S21 and S22). Moreover, the rejection rate can reach >99% toward various water-soluble organic dyes with a molecule size larger than 1.1 nm (Figure S23) and also >80% toward monovalent, divalent, and trivalent ions (i.e., Cl^–^, Na^+^, CO_3_^2+^, Gd^3+^, SO_4_^2+^) with hydrated ions diameters ranging from 0.6 to 1 nm (Figure S24, Tables S1 and S2). However, under the same conditions, the TpPa-1 membrane shows low rejections for some dyes such as acid fuchsin (29.2%), rose bengal (36.8%), and methylene blue (8.3%) and also exhibits low rejection for some salts such as NaCl (48.3%). The obtained pore-size distributions (Figure S25) by rejecting neutral solutes of polyethylene glycol (PEG) with different molecular weight verified the ultramicropores (about 0.44 nm) of the LA-α-CD-in-TpPa-1 membrane. In addition, the possibility of LA-α-CDα-CD-in-TpPa-1 instead being a mixed matrix COF membrane with α-CD inclusions can be rationally excluded based on the effective nanofiltration performance (Figures S23, S24, and S26). These results further indicate the compacted structure and narrowed pore size in the LA-α-CD-in-TpPa-1 membrane after incorporation of LA-α-CD. Despite a decline, the value of H_2_/CO_2_ separation selectivity keeps above 15 with the pressurization, and also the separation selectivity could be recovered to the previous value of about 30 after release of the pressure. These findings suggest the good pressure-resistant property of the host–guest LA-α-CD-in-TpPa-1 membrane.

Figure 3e,f illustrate the selectivity of H_2_/CO_2_ and H_2_/CH_4_ versus H_2_ permeance for our LA-α-CD-in-TpPa-1 membrane and other types of membranes (see the detailed comparison in Tables S3 and S4). In contrast to other membranes, the LA-α-CD-in-TpPa-1 membrane exhibits high values in terms of both permeance and selectivity, demonstrating an anti-trade-off phenomenon. The overall performance far exceeds the Robeson upper-bound limits for H_2_/CO_2_ and H_2_/CH_4_ mixtures,^68^ respectively, and belongs to the most powerful COF membranes. The excellent performance together with the good stability could also provide circumstantial evidence of fast and H_2_-preferential transport pathways within the linear α-CD polymer-tailored COF nanochannels.

To demonstrate the versatility of the pore-in-pore design concept, we prepared three other types of LA-α-CD-in-COF membranes (LA-α-CD-in-TBPa-1, LA-α-CD-in-TBBD, and LA-α-CD-in-TpBD membranes, as shown in Figures S27–S29) by using a similar synthesis protocol as for the LA-α-CD-in-TpPa-1 membrane. The gas permeation measurements were conducted at room temperature by using an equimolar H_2_/CH_4_ mixture as feed (Figure S30). It can be seen that all of the membranes exhibit a very good H_2_ permeance higher than 2800 GPU. The H_2_/CH_4_ separation selectivities of the LA-α-CD-in-TBPa-1 membrane, LA-α-CD-in-TBBD membrane, and LA-α-CD-in-TpBD membrane could reach 30.6, 20.6, and 21.5, respectively. Moreover, their comprehensive performances are also competitive and higher than most of the existing COF gas membranes without α-CDs,^29,30^ illustrating the potential and broad applicability of this pore-in-pore strategy.

### Molecular Dynamics Simulation and Transport Mechanism Analysis

**Figure 4:**
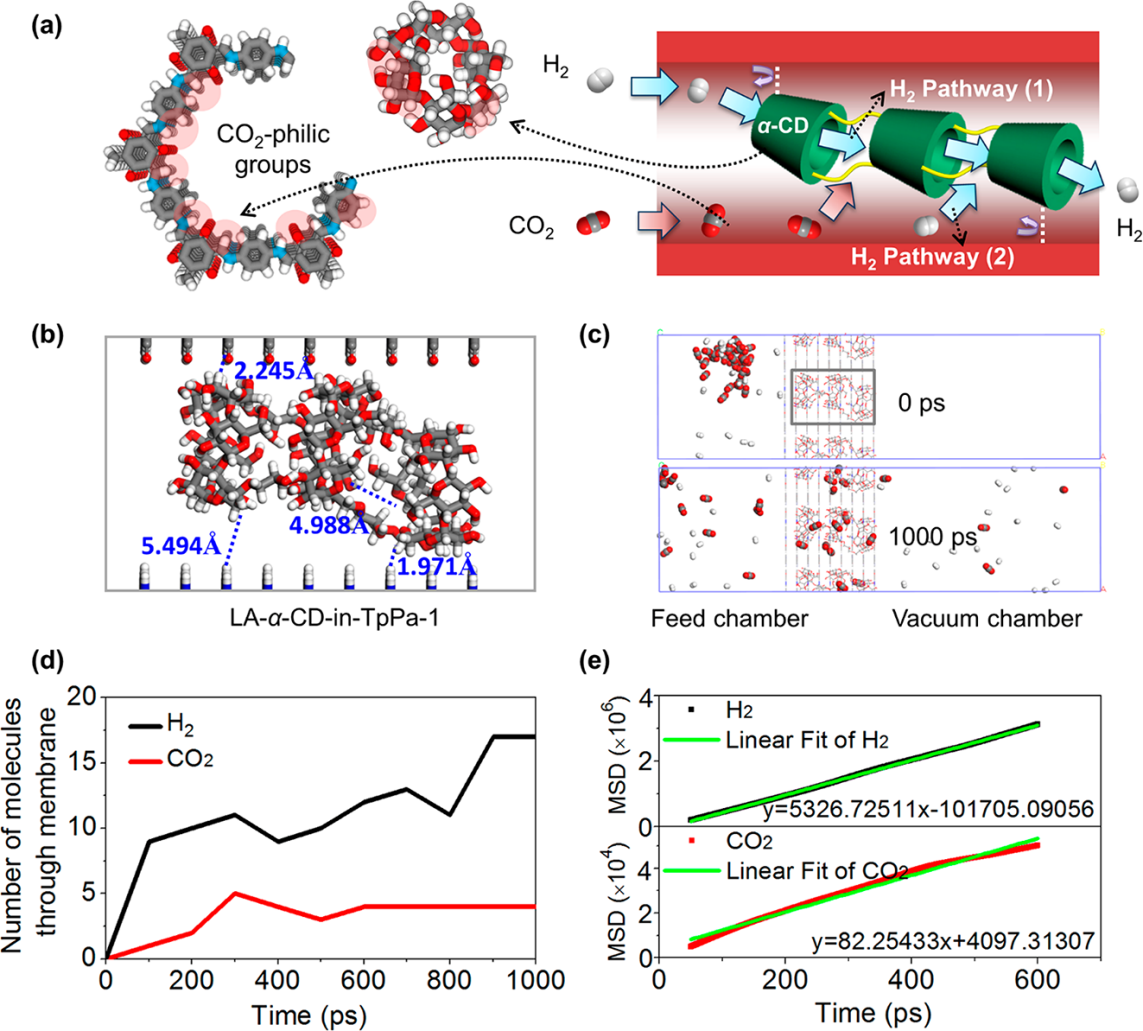
(a) Schematic illustration of the transport pathways of H_2_ and CO_2_ molecules through an LA-α-CD-in-TpPa-1 channel. (b) MD simulated molecular structure of an LA-α-CD-in-TpPa-1 channel. (c) Simulation system with snapshots at 0 and 1000 ps for the permeation of an equimolar H_2_/CO_2_ mixture (30 H_2_ and 30 CO_2_ molecules in the feed chamber) through the LA-α-CD-in-TpPa-1 membrane. (d) Number of gas molecules that passed through the LA-α-CD-in-TpPa-1 membrane in MD simulation as a function of simulation time for mixed-gas permeation. (e) Calculated mean square displacement (MSD) values simulation time for H_2_ and CO_2_.

Apparently, the engineering of pore-in-pore channels by packing of linear CD polymers contributes to the improvement in gas-separation performance of the COF membrane. To elucidate the separation mechanism on a microscopic scale, molecular dynamics (MD) simulations were carried out to investigate the permeation behavior of equimolar H_2_/CO_2_ through the starting TpPa-1 membrane and the LA-α-CD-in-TpPa-1 membrane (Figure S31). The simulations show two preferential transport pathways for H_2_ molecules in the LA-α-CD-in-TpPa-1 channels, (Figure 4a and b): Pathway (1) leads through the interior of encapsulated linear α-CD polymer, and pathway (2) is the gap between the pore wall and the LA-α-CD. The CO_2_ molecules, in contrast, are mainly adsorbed at the surface of the COF wall due to the CO_2_-philic groups (−NH–, C=O) and at the LA-α-CDs surface (−OH, C–O–C). These strong adsorptive interactions retard the transport of CO_2_ molecules and result in a low CO_2_ permeation, simultaneously narrowing this gap for the mobile H_2_ molecules. As shown in Figure 4c,d, 60% of the H_2_ molecules (18 out of 30 molecules) in the feed chamber could pass through the LA-α-CD-in-TpPa-1 membrane to the permeate chamber within 1000 ps, accompanied by only 13.3% of the CO_2_ molecules (4 out of 30 molecules), demonstrating a faster H_2_ permeation. Further, the H_2_ diffusion coefficient could be calculated from the linear fitting of the MSD in Figure 4e, which is about 65 times as large as the CO_2_ diffusivity. This finding signifies that the simulated separation selectivity is 1.8 times the experimental measurement (about 35). On the contrary, in an TpPa-1 channel without LA-α-CD (Figures S32 and S33), the transport of CO_2_ molecules at the pore wall is retarded. However, at the center of the pore channels the diffusion of CO_2_ molecules is not affected. In this case, a large proportion of CO_2_ molecules together with H_2_ molecules can pass through the membrane. As expected, 47% of the H_2_ molecules (14 out of 30 molecules) passed through but were accompanied by 43% of the CO_2_ molecules (13 out of 30 molecules) at the same time. Here, the calculated H_2_ diffusion coefficient is about 23 times larger than the CO_2_ one (Figure S33b), which is 1.6 times the corresponding experimental measurement (*D*(H_2_)/*D*(CO_2_) ≈ 14.5). However, a difference in performance between experimental measurement and MD simulation can be expected since the modeling structure cannot be strictly consistent with that of the real membrane structure on the macroscopic scale. For example, the low content of α-CD and the not strictly aligned ultramicropore structure will probably result in relatively low H_2_/CO_2_ selectivities in the experimental measurements. Despite this, the quotients of measured selectivity and simulated selectivity for the LA-α-CD-in-TpPa-1 membrane (∼1.8) and TpPa-1 membrane (∼1.6) are very close to each other. Meanwhile, the result of the simulated selectivity for the LA-α-CD-in-TpPa-1 membrane being higher than that for the TpPa-1 membrane is basically in agreement with the experimental measurements. Likewise, the H_2_ molecules permeate faster through the LA-α-CD-in-TpPa-1 channels along the same pathways mentioned above in the presence of CH_4_ molecules, as compared to the permeation process inside the TpPa-1 channels (Figures S34–S37). The simulations indicate that the performance improvement of the pore-in-pore membrane is mainly attributed to the competitive diffusion mechanism in the confined ultramicropore channels rather than to rigid size-sieving effects. Furthermore, these results imply that increasing the LA-α-CD content within the COF nanopores and maintaining the ordered structure of the host–guest confinement is crucial for the membrane discrimination accuracy for molecular–selective gas separations.

## Conclusions

We have developed a pore-in-pore strategy for packing linear α-cyclodextrin (α-CD) polymers into the COF nanochannels to engineer COF membranes with a matreshka-like pore structure suitable for gas separation. The formation of ultramicropore-in-nanopore channels retards the diffusion of bulky molecules such as CO_2_ and CH_4_ but renders preferential transport pathways for the H_2_ molecules. Owing to the competitive diffusion mechanism in the confined ultramicropore channels, the linear assembly-α-CD-in-TpPa-1 (from the reaction of 1,3,5-triformylphloroglucinol (Tp) and *p*-phenylenediamine (Pa-1)) membrane displays an ultrahigh H_2_ permeance and significantly enhanced separation selectivity for gas mixtures as compared to the starting TpPa-1 membrane without α-CD inclusion. The excellent overall performances combined with a high stability recommend the pore-in-pore COF membranes for advanced H_2_ purification and production. In consideration of a certain generalizability, this research complements the existing design strategies of ultramicroporous COF membranes and could facilitate their advancement in the field of precise molecular separations. The preparation of linear arrangements of guest species in the nanochannels of COFs is also of interest for the preparation of functional host–guest materials.

## Experimental Section

### Experimental Materials

All chemicals and materials were used as received without further purification: *p*-phenylenediamine (Pa-1) (>99%, Sigma), benzidine (BD) (98%, Sigma), 1,3,5-triformylphloroglucinol (Tp) (>96%, Ark), 1,3,5-triformylbenzene (TB) (96%, Acros), acetic acid (AR, 36%, Roth), α-cyclodextrin (α-CD, ≥98%, Sigma), 3-aminopropyltriethoxysilane (APTES, 99%, Sigma), epichlorohydrin (ECH, ≥99%, TCI), ethanol (>99%, Acros), sodium hydroxide (NaOH, ≥98%, Sigma), hydrochloric acid (HCl, 36%, Sigma), polyethylene glycols with various molecular weights (PEG, 200, 400, 600, 800, 1000, 2000, 4000 Da, Macklin). Sodium chloride (NaCl, AR, 99.5%), sodium sulfate (Na_2_SO_4_, AR, 99%), sodium carbonate (Na_2_CO_3_, AR, 99%), and gadolinium chloride (GdCl_3_, AR, 99%) were provided by Sinopharm Chemical, China. Dyes including chrome black T, methyl blue, acid fuchsin, congo red, rose bengal, and methylene blue (MEB) were supplied by Shanghai Macklin Biochemical Technology, China. Porous asymmetric α-Al_2_O_3_ disks (with a 70 nm pore-top layer, 18 mm in diameter and 1 mm in thickness) as substrates were purchased from Fraunhofer IKTS, Germany.

### Synthesis of LA-α-CD-in-COF Membranes

The porous α-Al_2_O_3_ substrate was activated by using HCl aqueous solution (1 M), and then the surface was modified with APTES (2 mM in toluene) at 110 °C for 2 h under argon. The LA-α-CD-in-TpPa-1 membrane was synthesized via an α-CD-embedded interfacial polymerization followed by a linear assembly (LA). First, 24 mg of Pa-1 and 20 mg (or 10 mg, 30 mg, 40 mg) of α-CD were dissolved into 12 mL of ultrapure water, and then 2 mL of acetic acid aqueous solution (3 M) was added to form the solution A. After that, 31.5 mg of Tp was dissolved into 14 mL of toluene to form solution B. It should be noted that both solution A and solution B were prepared under argon atmosphere. Before interfacial polymerization, the amino-α-Al_2_O_3_ substrate was thoroughly immersed into solution A until it was saturated. Subsequently, the amino-α-Al_2_O_3_ substrate was fixed in between solution A and solution B with a homemade device, which was then horizontally placed at 120 °C for 72 h to allow the growth of the α-CD-in-TpPa-1 layer onto the amino-α-Al_2_O_3_ substrate. After cooling, the formed α-CD-in-TpPa-1 membrane was washed with water and ethanol and dried at 120 °C overnight. Finally, the α-CD-in-TpPa-1 membrane was soaked into the prepared ECH alkaline solution (20 mg in 14 mL of NaOH (25%, g/mL) solution) at 50 °C for 8 h to cross-link the packed α-CDs. This process is named as LA in this study. The LA-α-CD-in-TpPa-1 membrane was obtained after a thorough rinsing by ultrapure water. It should be pointed out that the α-CD used was structurally stable in the weak acidic environments during the interfacial polymerization, and the membrane was stable in the alkaline environments during the cross-linking of the packed α-CDs because of the short immersion time.

For comparison, the TpPa-1 membrane without α-CD and the α-CD-in-TpPa-1 membrane were synthesized by using a similar procedure as mentioned above. Three other types of LA-α-CD-in-COF membranes were prepared by using a similar protocol as for the LA-α-CD-in-TpPa-1 membrane. The main difference is that the LA-α-CD-in-TBPa-1 membrane was synthesized from 24 mg of Pa-1, 20 mg of α-CD, and 24 mg of TB; the LA-α-CD-in-TBBD membrane was synthesized from 27.6 mg of BD, 20 mg of α-CD, and 16 mg of TB; the LA-α-CD-in-TpBD membrane was synthesized from 27.6 mg of BD, 20 mg of α-CD, and 21 mg of Tp.

### Characterization

Micromorphologies of the membranes and powdered samples were observed by using a JEOL JSM-6700F instrument with a cold field emission gun operating at 2 kV and 10 mA. Before measurement, all samples were coated with a 15 nm thick gold layer in vacuo to reduce the charging effects. Energy-dispersive X-ray spectroscopy (EDXS) mapping and elemental analysis of the membrane cross section were conducted on the scanning electron microscopy (SEM) at 15 kV, 10 mA and 15 mm lens distance. For transmission electron microscopy (TEM) measurements, a small drop of aqueous solution of mashed membrane, LA-α-CD, or α-CD was dripped on an ultrathin carbon support film and dried, and then the specimen was observed with a JEM2100F microscope. X-ray diffractometer (XRD) patterns were recorded on a Bruker D8 Advance diffractometer (Cu Kα X-ray radiation, λ = 1.54 Å) at room temperature, and each XRD pattern was acquired from 3° to 35° of the diffraction angles at a rate of 0.01° s^–1^, a voltage of 40 kV, and current of 40 mA. X-ray photoelectron spectroscopy (XPS) spectra were recorded on a Thermo Scientific K-Alpha+ spectrometer using Al Kα radiation as the energy source at a voltage of 15 kV and current of 15 mA. The pressure in the instrumental chamber was about 5 × 10^–9^ mbar. The binding energies were calibrated on C 1s (284.8 eV). Attenuated total reflectance-Fourier transformed infrared spectra (ATR-FTIR) in a wavenumber region of 400–4000 cm^–1^ with a resolution of 0.4 cm^–1^ were obtained by using a spectrometer (Agilent Technologies Cary 630 FTIR). The ATR-FTIR spectrum of the membrane samples was obtained from the powders shaved off from the selective layer on the α-Al_2_O_3_ substrate. Raman spectra were acquired by a Horiba LabRAM HR Evolution instrument using an Ar+ laser at 514.5 nm. Fluorescence imaging was recorded on an Olympus IX71 microscope at an excitation wavelength of 546–560 nm. Before measurement, membrane samples were submerged in an aqueous rhodamine B solution and subjected to the treatments including rinsing with water and drying. N_2_ adsorption–desorption measurements were performed at 77 K on a Micromeritics ASAP2460 surface area and pore distribution analyzer instrument. Before the adsorption experiments the powdered samples were vacuum degassed at 120 °C for 10 h. The isotherms were analyzed by using the Brunauer–Emmett–Teller method and the t-plot micropore volume method.

### Gas-Separation Measurement

The prepared membrane was placed inside a laboratory-made gas-permeation apparatus (Figure S13) sealed with rubber O-rings. N_2_ was used as the sweep gas set at 50 mL min^–1^ during the measurement process, and the pressures at both sides were kept at about 1 bar. It should be noted that the feed pressure is always a little bit larger than the permeate side to avoid the possibility of back flow of the sweep gas. Before gas permeation, an on-stream activation was carried out at 393 K to get rid of potential solvent molecules inside the pores of the membrane by using an equimolar H_2_/CO_2_ mixture as the feed. For the measurement of single gas permeation, both feed and sweep flow rates were set at 50 mL min^–1^. For the measurement of mixed-gas permeation, a series of equimolar (1:1) binary gas mixture including H_2_/CO_2_, H_2_/CH_4_, H_2_/C_3_H_6_, and H_2_/C_3_H_8_ were applied to the feed side of the membrane, and the feed flow rate of each gas was kept constant at 25 mL min^–1^. A calibrated gas chromatograph (HP 6890B) was used to detect the component concentration on the permeate side of the membrane when the measurement system ran stable.

The permeance of component *i* (*P*_*i*_) was calculated as follows (eq 1)
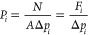
1where *N*_*i*_ represents the permeation rate of component *i* (mol s^–1^), *A* is the effective membrane area (m^2^), and Δ*p*_*i*_ is the partial pressure difference of component *i* (Pa). *F*_*i*_ denotes the molar flux of component *i* (mol m^–2^ s^–1^). Permeance of each membrane was calculated by the average of five data points. The GPU is the unit of gas permeance (1GPU = 3.3928 × 10^–10^ mol m^–2^ s^–1^ Pa^–1^).

The ideal selectivity of the membrane was calculated as the quotient of the permeance of component *i* (*P*_*i*_) divided by the permeance of component *j* (*P*_*j*_) in the single-gas permeation. The real selectivity (α_*i,j*_) of an equimolar binary gas mixture (or separation factor) was calculated as follows (eq 2)
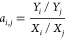
2where *x* and *y* denote the molar fractions of the corresponding component *i, j* in the feed and permeate side, respectively.

### Nanofiltration Measurement

Dye and salt rejection as well as water permeation were carried out by placing the membrane into a cross-flow nanofiltration apparatus (Figure S13). A dye aqueous solution (100 mg/L) or a salt aqueous solution (1000 mg/L) as the feed was circulated by using a plunger pump, and the operating pressure was set at 4 bar. The measurement began when the filtration system reached a steady state after a period of operation. The water flux (L m^–2^ h^–1^) and permeance (L m^–2^ h^–1^ MPa^–1^) were calculated by normalizing the permeate volume collected during the time *t*. The concentration of dyes and salts in the feed (*C*_*if*_) and permeate (*C*_*ip*_) was monitored by a UV–vis detector (UV BlueStar A) and a conductivity meter (KEDIDA CT3030), respectively. Accordingly, the rejection (*R*_*i*_, %) of the dyes or salts was calculated as follows (eq 3):
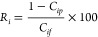
3

### Molecular Dynamics (MD) Simulations

The eclipsed atomic structure of TpPa-1 (*a* = *b* = 22.556 Å) composed of nine-layered nanosheets with a thickness of approximately 2.7 nm along the *z*-axis was conducted as shown in Figure S31a. Based on the most possible case of statistically channel-type linear arrangement that we have analyzed, the atomic structure of LA-α-CD-in-TpPa-1 is built by incorporating a linear α-CD polymer consisting of three cross-linked α-CDs into one nanochannel of the TpPa-1, as illustrated in Figure S31b. To some extent, it represents the elementary mass transfer unit in the 1.5 μm-thick LA-α-CD-in-TpPa-1 membrane. MD simulation was carried out by a Materials Studio software 6.0 package with a COMPASS force field.^69,70^ A typical simulation box with a dimension of 45.1 Å × 45.1 Å × 139.4 Å was established and separated into two chambers by the TpPa-1 layer or LA-α-CD-in-TpPa-1 layer from the middle. An equimolar mixture of H_2_/CO_2_ or H_2_/CH_4_ (30 molecules for each component) was added to the left chamber, and a vacuum was applied on the right. An NVT (constant particle number, volume and temperature) ensemble was employed for simulation, and system optimizing was implemented before diffusion simulation. The initial velocities were random, and the Andersen thermostat was employed to maintain a constant simulation temperature of 298.0 K. The MD simulation was performed for 1 ns with a time step of 1 fs using the Forcite module. The diffusion coefficient is related to the mean square displacement (MSD) and simulation time. The diffusion coefficients of the H_2_, CO_2_, and CH_4_ were calculated from the slope of the straight line fitted from the MSD versus simulation time (the value of slope divided by 6 is the diffusion coefficient).

## Supporting Information Available

Additional characterizations including SEM, Raman, fluorescence, XRD, TEM, ATR-FTIR, structural analysis of the membrane and powder samples; schematics of gas-permeation and cross-flow apparatuses; additional MD simulation results, gas separation performance, dyes and ion rejection performance; diameter of hydrated ions and molecule size of water-soluble dyes, detailed information on the gas-permeation data of various membranes (PDF)
