# Supplementary material for: Pore-in-Pore Engineering in a Covalent Organic Framework Membrane for Gas Separation
Source: ACS Nano. 2023 Apr 7;17(8):7584–94. doi: 10.1021/acsnano.2c12774 (PMC10134499; doi:10.1021/acsnano.2c12774)
Supplement: Supplementary file 1 — nn2c12774_si_001.pdf [file nn2c12774_si_001.pdf]

Supporting Information

Pore-in-Pore Engineering in a Covalent Organic Framework Membrane for Gas Separation

Hongwei Fan,[†,‡] *Haoran Wang,*[†] *Manhua Peng,*[§] *Hong Meng,*[†] *Alexander Mundstock,*[‡] *Alexander Knebel,*[#] *and Jürgen Caro*[‡,*]

[†]College of Chemical Engineering, Beijing University of Chemical Technology, Beijing 100029, PR China

[‡]Institute of Physical Chemistry and Electrochemistry, Leibniz Universität Hannover, Callinstraße 3A, 30167 Hannover, Germany

[§]Key Laboratory of Power Station Energy Transfer Conversion and System, Ministry of Education, School of Energy Power and Mechanical Engineering, North China Electric Power University, Beijing 102206, PR China

[#]Otto Schott Institute of Materials Research, Friedrich Schiller University Jena, Fraunhoferstraße 6, 07743 Jena, Germany

*E-mail: juergen.caro@pci.uni-hannover.de

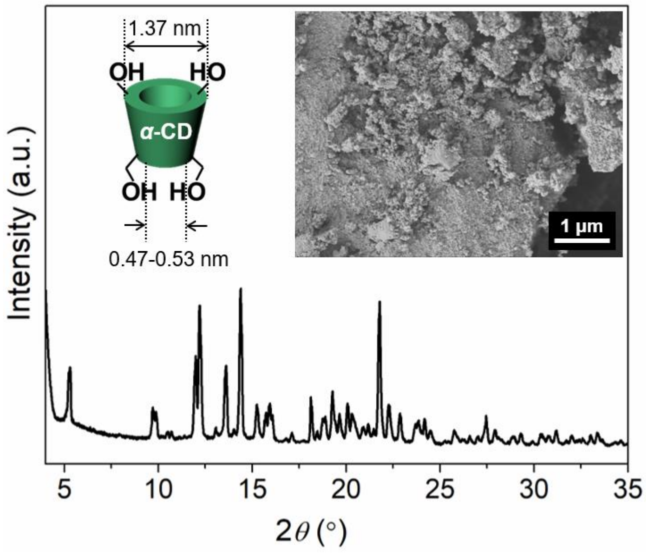

Figure S1. XRD patterns with inserted schematic structure and SEM image of *α*-CD used in this study.

The XRD patterns reveal the cage-type structures of *α*-CD, which is in agreement with the literature findings.[S1-S3]

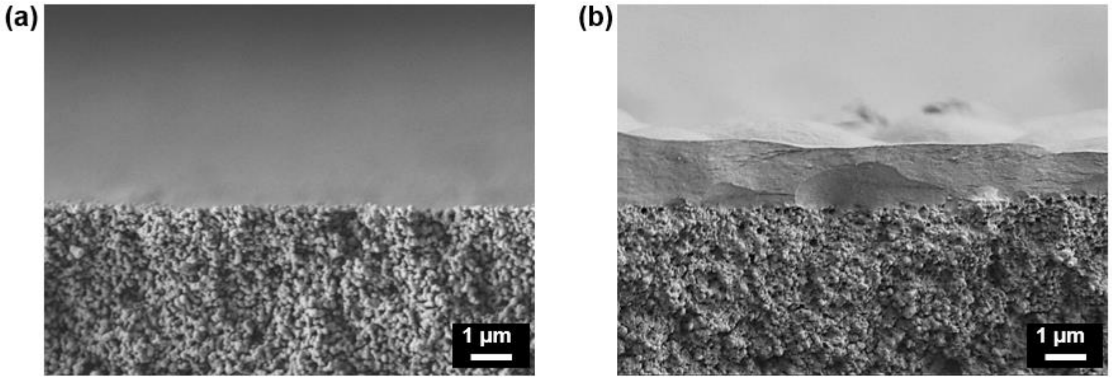

Figure S2. Cross-sectional SEM images of (a) *α*-Al_2O_3 substrate and (b) LA-*α*-CD-in-TpPa-1 membrane.

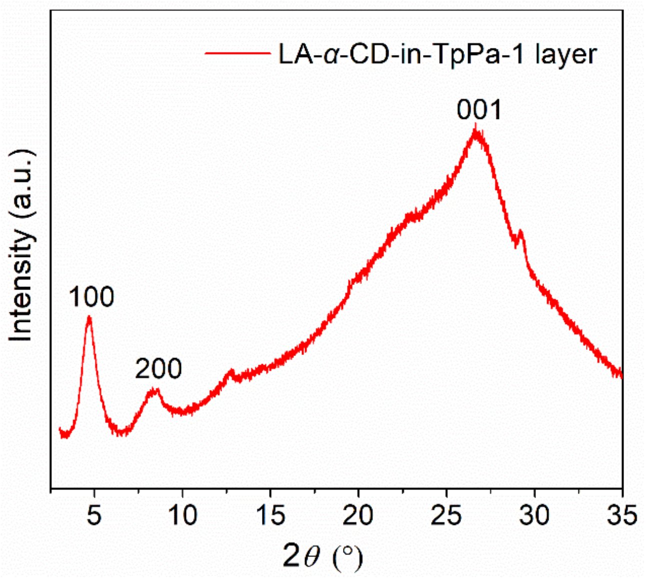

Figure S3. XRD patterns of the self-supporting LA-*α*-CD-in-TpPa-1 layer synthesized by *in situ* interface polymerization.

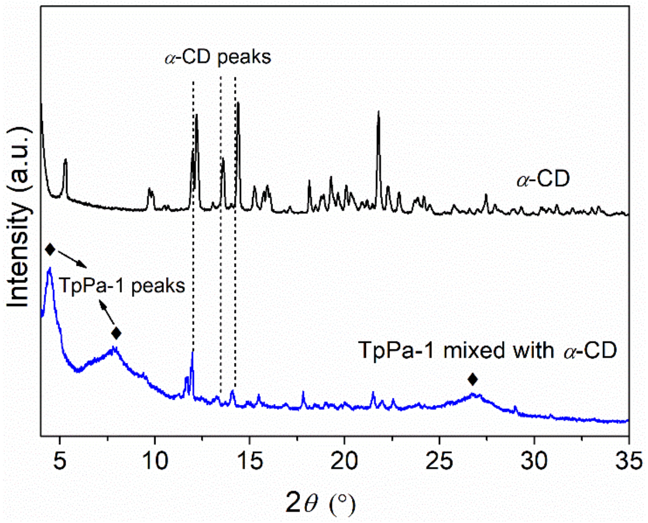

Figure S4. XRD patterns of the TpPa-1 powders mixed with *α*-CD (about 10%, close to the calculated content from XPS).

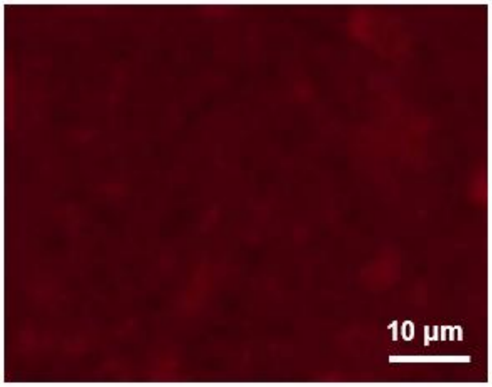

Figure S5. Cross-sectional fluorescence image of the LA-α-CD-in-TpPa-1 selective layer.

To satisfy the measurement requirement, the self-supporting LA-α-CD-in-TpPa-1 layer with a much thicker thickness (hundreds of microns) was prepared for cross-sectional fluorescence characterization.

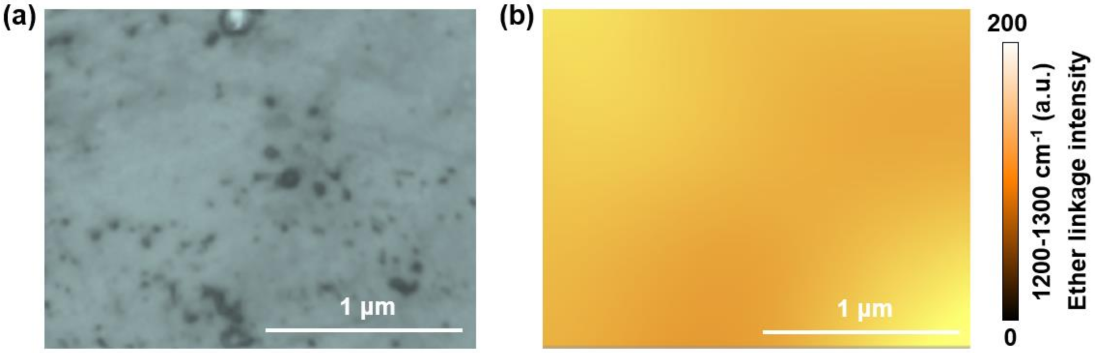

Figure S6. (a) Cross-sectional optical microscopy image of the LA-α-CD-in-TpPa-1 layer for Raman mapping. (b) Raman mapping ranging from 1200 to 1300 cm^{-1} over the area in (a).

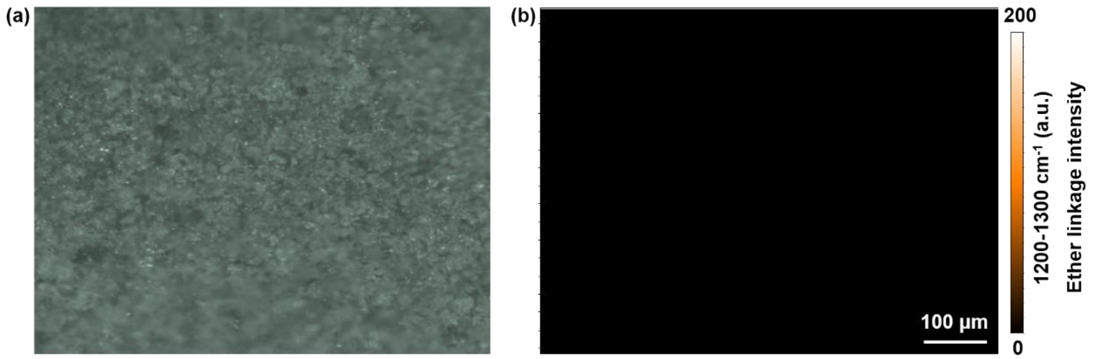

Figure S7. (a) Optical microscopy image of the TpPa-1 powders (compacted to thin film before measurement) for Raman mapping. (b) Raman mapping ranging from 1200 to 1300 cm^{-1} over the area in (a).

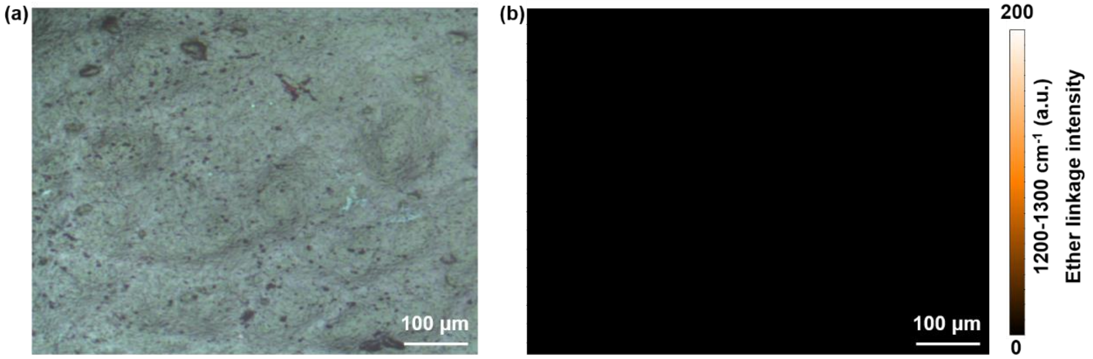

Figure S8. (a) Surface optical microscopy image of the TpPa-1 membrane for Raman mapping. (b) Raman mapping ranging from 1200 to 1300 cm^{-1} over the area in (a).

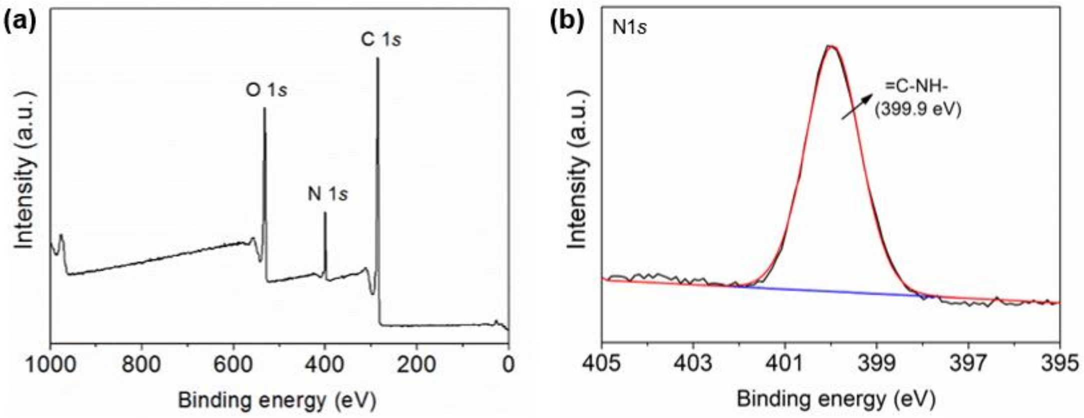

Figure S9. (a) Typical XPS survey spectra of LA-α-CD-in-TpPa-1. High-resolution XPS spectra of deconvoluted C1*s* (b) and N1*s* (c) in LA-α-CD-in-TpPa-1.

The α-CD volume fraction (vol%) could be estimated based on the C-O-C/C=O atomic (molar) ratio measured by XPS on the LA-α-CD-in-TpPa-1 layer. Based on the chemical formula of α-CD ($C_{36}H_{60}O_{30}$) and TpPa-1 ($C_{18}H_{12}N_3O_3$), and their densities of ~0.6 g/cm^3 and ~0.8 g/cm^3, respectively), the calculated loading of α-CD in the COF matrix is no more than 11.8 vol%, considering the LA.

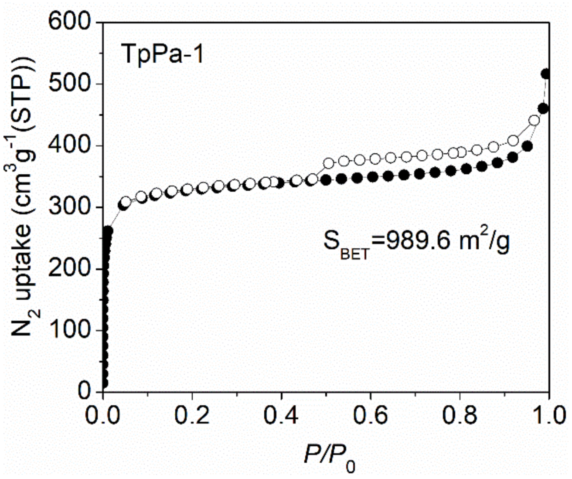

Figure S10. Nitrogen adsorption and desorption curves and corresponding BET surface area value (inset) of the synthesized TpPa-1 powders (adsorption, closed; desorption, open symbols).

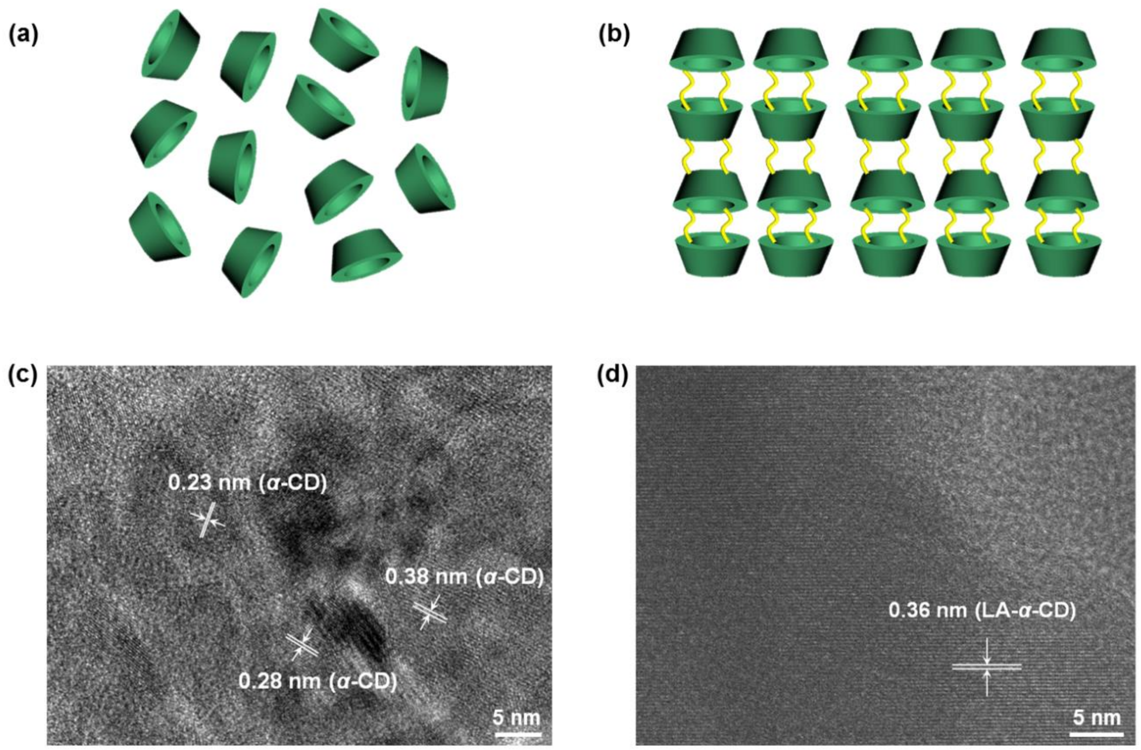

Figure S11. Schematic representation of the α-CD molecules within their crystal of (a) cage-type and (b) channel-type structures. High-resolution TEM images with marked lattice fringes of (a) LA-α-CD and (b) α-CD.

Generally, there are two types of crystal structures for α-CD molecules, including cage-type structure (Figure S11a) and channel-type structure (Figure S11b). In the cage structure, α-CD molecules are arranged in a "herringbone" or "brick" fashion, and both ends of the cavity are blocked off by adjacent α-CDs to form isolated cages.[S4,S5] In the channel-type structure, the "dimer units" formed by two neighboring α-CDs (head-to-head or head-to-tail orientation) are linearly stacked to form an "infinite" column in the crystal. In this work, for the primitive α-CD without crosslinking, a cage-type structure has been confirmed from the XRD patterns (Figure S1), and in this case, there are several types of the lattice fringes observed from the

corresponding high-resolution TEM image (Figure S11c). It was reported that the channel-type α-CD structure could be formed by various ways such as threading with a linear guest (*i.e.*, PEG), and crosslinking with ECH.[S6-S8] From high-resolution TEM image of LA-α-CD (Figure S11d), the lattice fringe becomes more ordered and is different from that of the α-CD without crosslinking, suggesting the possible formation of channel-type structures.

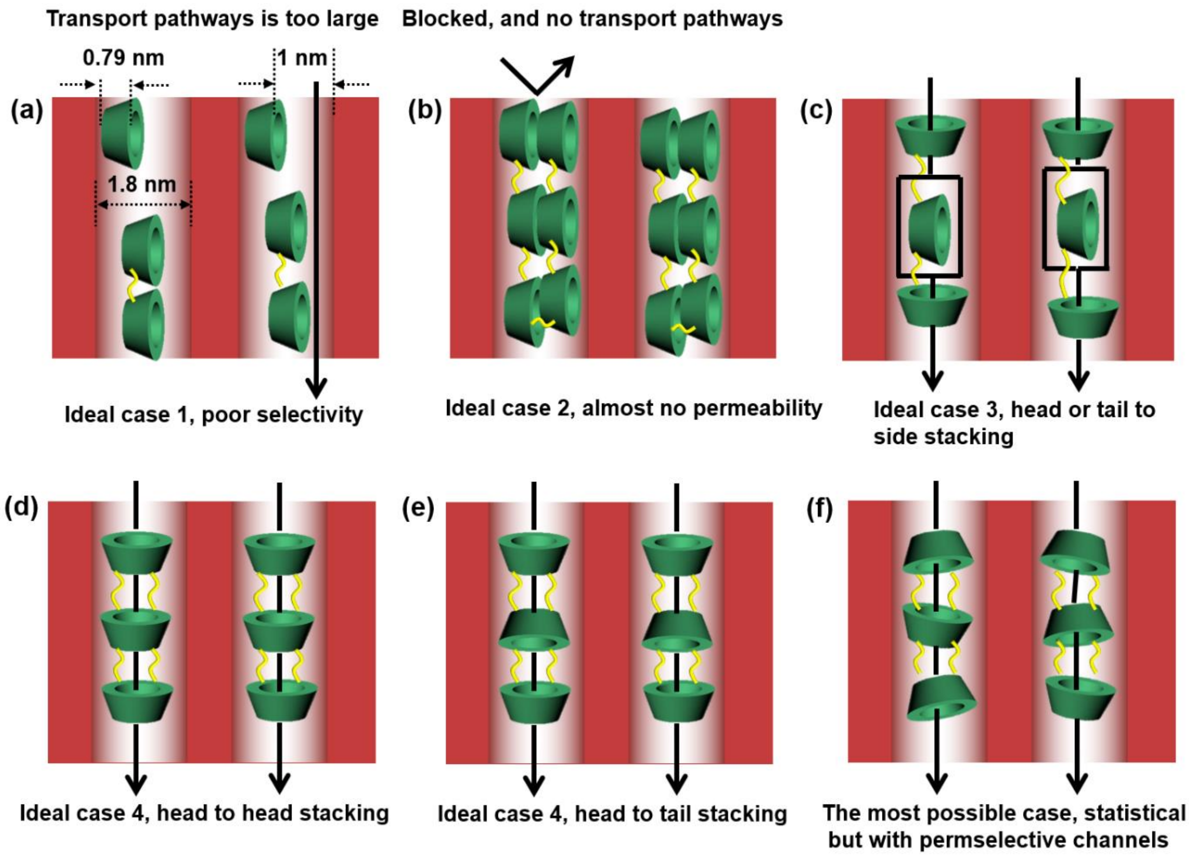

Figure S12. Analysis of possible configuration and orientation of linear α-CD polymers in COF nanochannels.

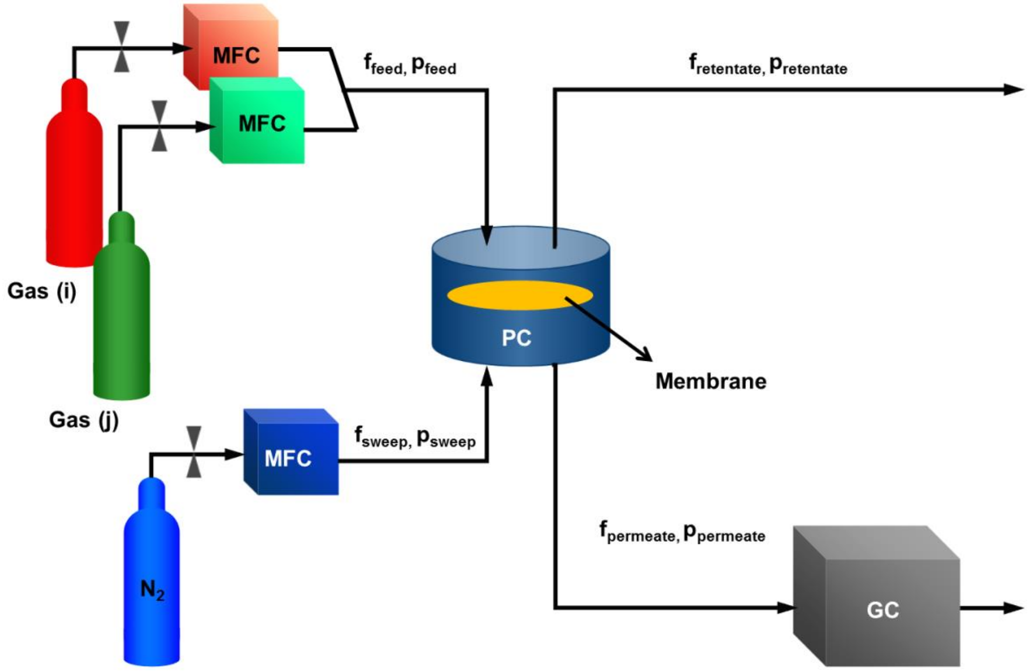

Figure S13. Measurement setup/equipment for single gas and mixed-gas permeations. MFC: mass flow controller; PC: permeation cell with mounted membrane; GC: gas chromatograph; f: volumetric flow rate; p: pressure.

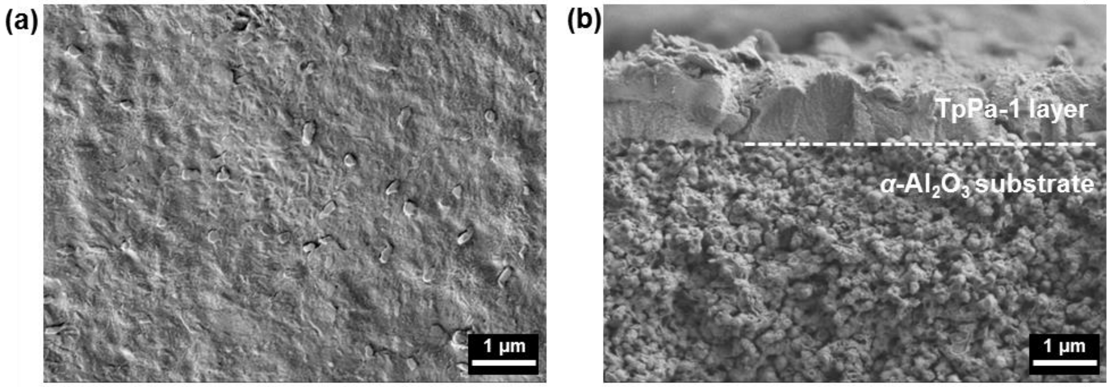

Figure S14. (a) Surface and (b) cross-sectional SEM images of the prepared TpPa-1 membrane.

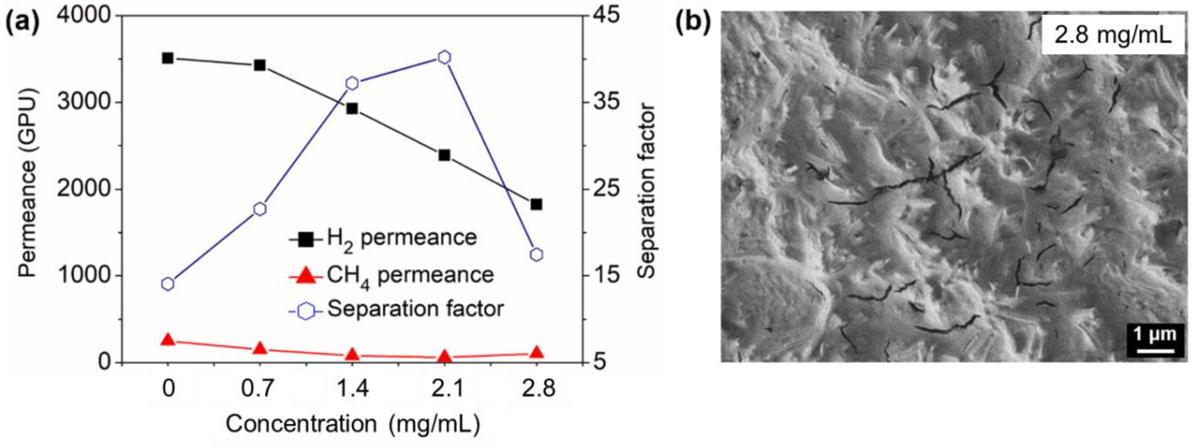

Figure S15. (a) The effect of α-CD concentration in the precursor solution on the H_2/CH_4 separation factor and H_2 permeances of the resulting LA-α-CD-in-TpPa-1 membrane. (b) Surface SEM image of the LA-α-CD-in-TpPa-1 membrane synthesized from an α-CD concentration of 2.8 mg/mL.

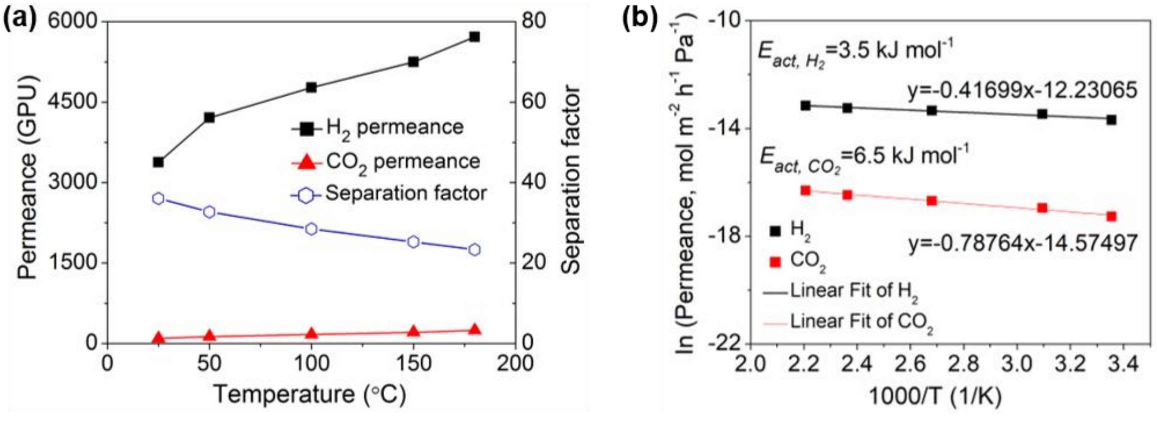

Figure S16. (a) H_2/CO_2 separation factor and H_2 permeances of the LA-α-CD-in-TpPa-1 membrane as function of the temperature at 1 bar. (b) Arrhenius temperature dependence of H_2 and CO_2 permeance through the LA-α-CD-in-TpPa-1 membrane.

It can be seen that the separation factor for H_2/CO_2 mixtures gradually decreases from 35.1 to 23.3 when the test temperature was increased from room temperature to 180 °C (Figure S16a). This could be reasonably explained by the diffusion activation energy (E_{diff}) for H_2 and CO_2, which is related to the activation energy ($E_{act,i}$) and adsorption heat (Q_{st}) (Eq. 1, as follows):

$$E_{act,i} = E_{diff} - Q_{st} \qquad (1)$$

As shown in Figure S16b, the ln (P_i) versus 1/T displays a linear correlation, so the $E_{act,i}$ can be calculated from the slope of the Arrhenius equation,[S9] and the calculated $E_{act,CO2}$ value is 6.5 kJ mol^{-1} higher than that of the $E_{act,H2}$ (3.5 kJ mol^{-1}). The adsorption heat of CO_2 is relatively high compared to that of the H_2, considering the CO_2-philic groups (-NH-, -OH, e.g.) in COF and α-CD, which have stronger adsorption for CO_2 molecules, and therefore the E_{diff} of CO_2 is certainly larger than the H_2. This demonstrates much more activated diffusion of CO_2 through the LA-α-CD-in-TpPa-1 membrane at higher temperature, thereby leading to a declining trend in separation factor for H_2/CO_2 mixture.

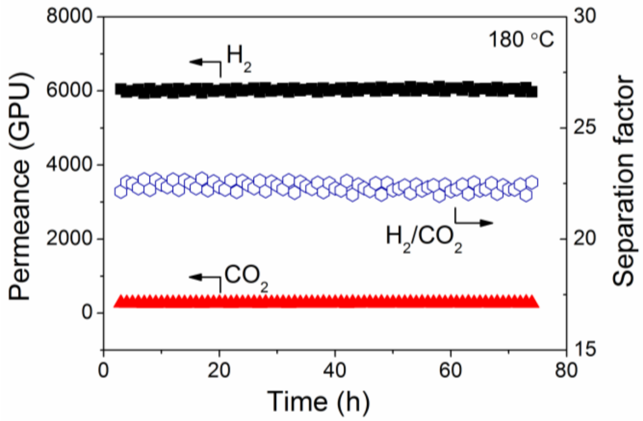

Figure S17. Long-term tests of LA-α-CD-in-TpPa-1 membrane for equimolar gas mixture of H_2/CO_2 at 453 K (180 °C) at 1 bar.

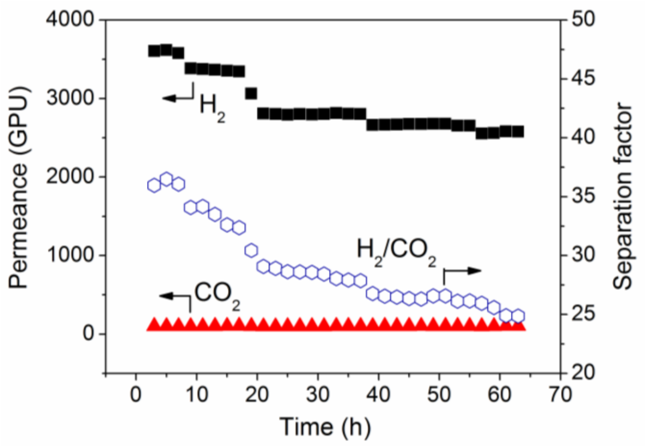

Figure S18. Long-term tests of α-CD-in-TpPa-1 membrane (without crosslinking/LA) for equimolar gas mixture of H_2/CO_2 at room temperature (25 °C) at 1 bar.

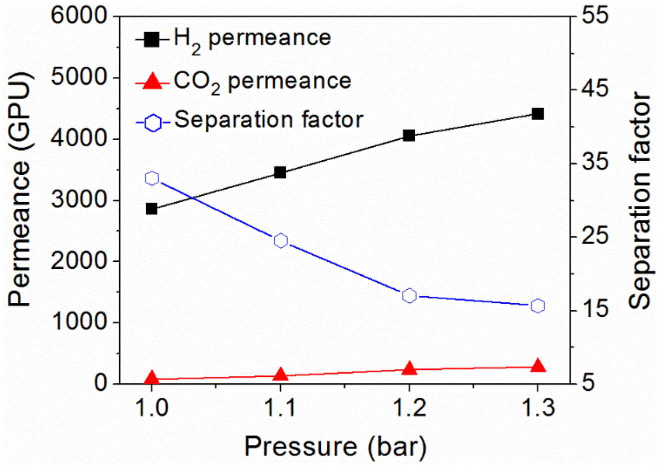

Figure S19. H_2/CO_2 separation factor and H_2 permeances of the LA-α-CD-in-TpPa-1 membrane as function of the pressure.

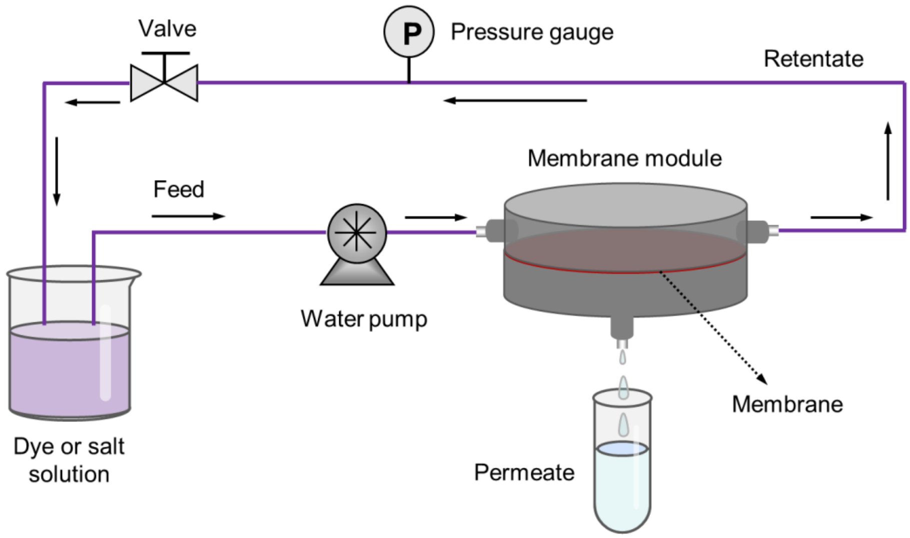

Figure S20. Schematic of experimental apparatus for cross-flow nanofiltration process.

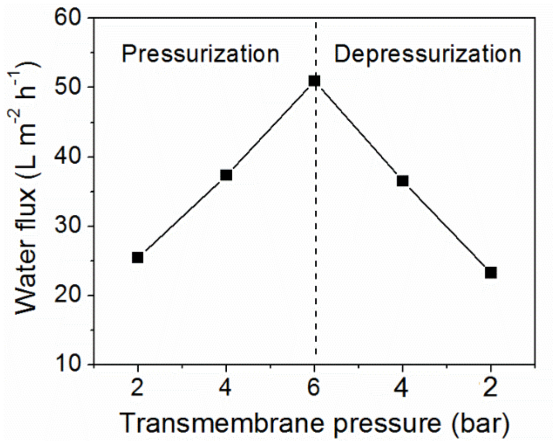

Figure S21. Dependence of water flux on transmembrane pressure for LA-α-CD-in-TpPa-1 membrane.

As shown in Figure S21, the water flux of the LA-α-CD-in-TpPa-1 membrane shows an linear relation to the transmembrane pressure whether in the pressurization (feed pressure increased from 2 to 6 bar) or depressurization (feed pressure decreased from 6 to 2 bar) process.

This result illustrates that the pore-in-pore transport nanochannels of the membrane do not collapse even at a high external pressure of 6 bar.

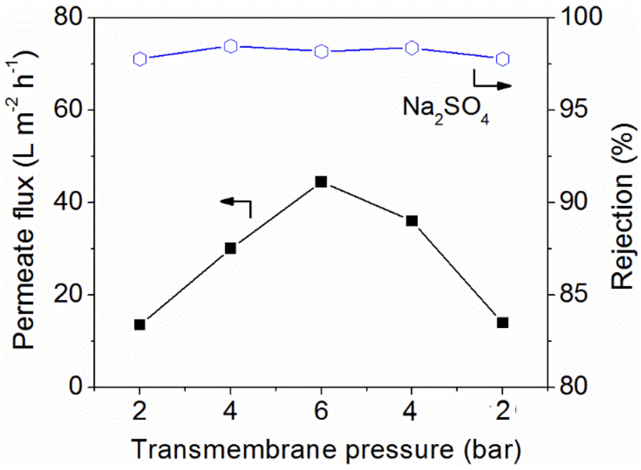

Figure S22. Dependence of permeate flux and Na_2SO_4 rejection on transmembrane pressure for LA-α-CD-in-TpPa-1 membrane.

When a Na_2SO_4 solution was used as the feed in the corss-flow nanofiltration, with the changes of feed pressure (increasing from 2 to 6 bar and then decreasing from 6 to 2 bar), the water permeate flux also exhibited a similar linear relation. During the whole process, the Na_2SO_4 rejection rate of the LA-α-CD-in-TpPa-1 membrane remained above 98%, indicating the potential of pressure resistance. Moreover, this result also suggests the possible reactions between ECH and non-reacted COF monomers (-OH, -NH_2), which immobilized LA-α-CD onto the TpPa-1 pore walls and made the unique ultramicropore-in-nanopore structure more stable in the aqueous phase.

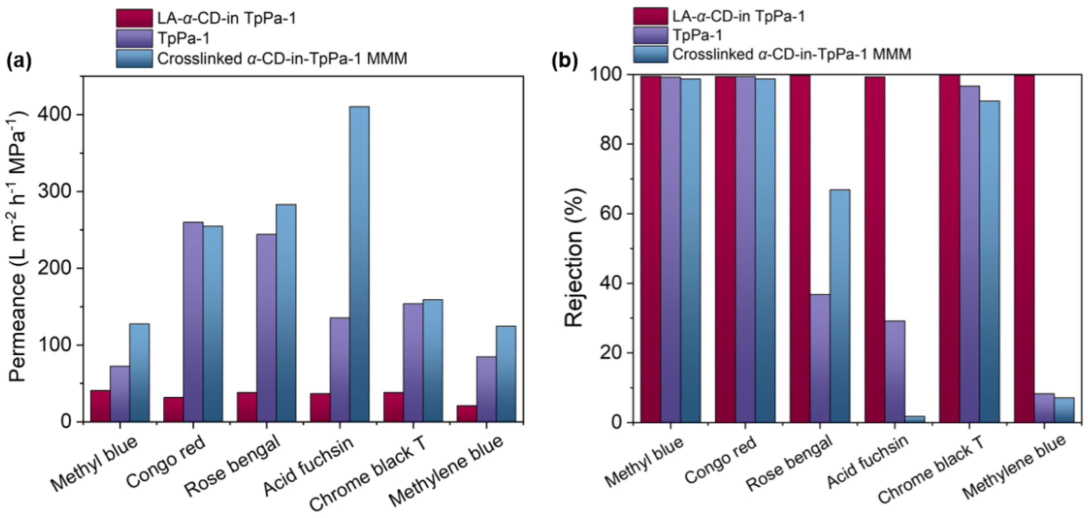

Figure S23. (a) Water permeance and (b) rejections of the LA-*α*-CD-in-TpPa-1 membrane, TpPa-1 membrane and corsslinked *α*-CD-in-TpPa-1 MMM for various dyes.

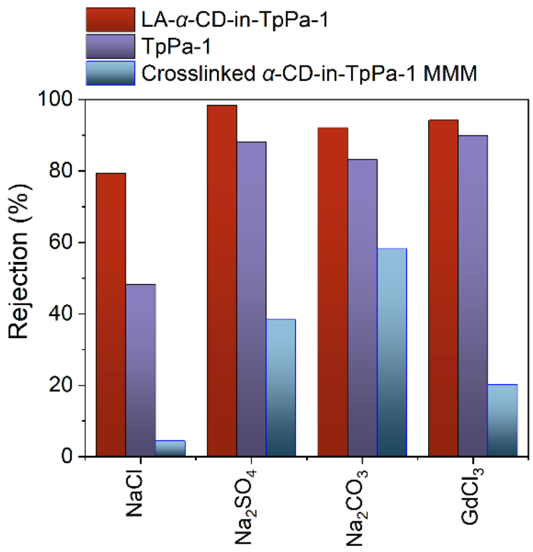

Figure S24. Salt rejection performance of the LA-*α*-CD-in-TpPa-1 membrane.

From Figure S24, the LA-*α*-CD-in-TpPa-1 membrane dispays excellent rejection rate of >99% towards various water-soluble organic dyes with a molecule size larger than 1.1 nm.

Moreover, the membrane can also retain NaCl (about 80%), Na_2SO_4 (98%), Na_2CO_3 (93%), $GdCl_3$ (95%), as shown in Figure S24. Considering that the hydrated diameter of different ions ranged from 0.6 nm to 1 nm, the high salt rejection rates indicate no defects and compactness of the LA-α-CD-in-TpPa-1 membrane.

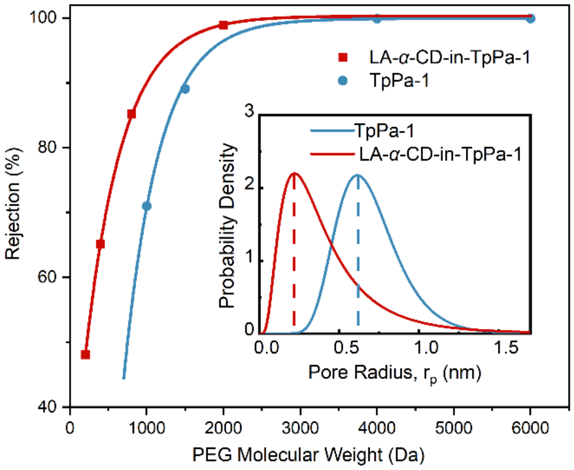

Figure S25. MWCO and pore-size distributions of TpPa-1 membrane and LA-*a*-CD-in-TpPa-1 membrane measured by rejecting PEG with different molecular weight.

Detailed measurements of MWCO, pore size and pore-size distribution of membrane: PEGs with molecular weights ranging from 200-6000 Da were selected to evaluate the rejection performances of membranes by nanofiltration. The concentration of each PEG in aqueous solution was 100 ppm. The concentration of PEG in the feed and filtrate was measured by using UV-barium chloride method. Before the absorbance test, the 0.5 ml of sample was taken and diluted 10 times with pure water to 5 ml of solution. Then 1 ml of 0.05 M iodine standard solution and 1.2 ml of 5% barium chloride solution were added. After about 10 minutes, the UV absorbance measurement was performed at a wavelength of 610 nm.

When PEG retention rate is 90%, the corresponding molecular weight is referred as the MWCO. The average pore size and pore distribution curve of membrane can be calculated according to the probability density function (PDF) equation (2), which is based on the premise that there is no spatial or hydrodynamic interaction between the membrane and the neutral molecules.[S10] The PDF equation is

$$f_R(r_p) = \frac{dR}{dr_p} = \frac{1}{r_p \ln \sigma_g \sqrt{2\pi}} exp(\frac{-(\ln r_p - \ln \overline{r_p})^2}{2(\ln \sigma_g)^2}) \quad (2)$$

Where, σ_g is calculated from the geometric standard deviation of PDF curve, which is defined as the ratio of the radius of the solute molecule with a rejection rate of 83.14% to the radius of the solute molecule with a retention rate of 50%. $\overline{r_p}$ represents the average pore size of the membrane, which is expressed as the Stokes radius of the solute molecule at 50% rejection.

The Stokes radius (r_s) of a molecule is calculated as:

$$ln(r_s) = -1.4962 + 0.4654\, ln(M_W) \quad (3)$$

M_W is the molecular weight of neutral solute molecule. The relationship between PEG molecular radius and M_W is shown as follows:

$$r_s = 16.73 \times 10^{-2} \times Mw^{0.557} \quad (4)$$

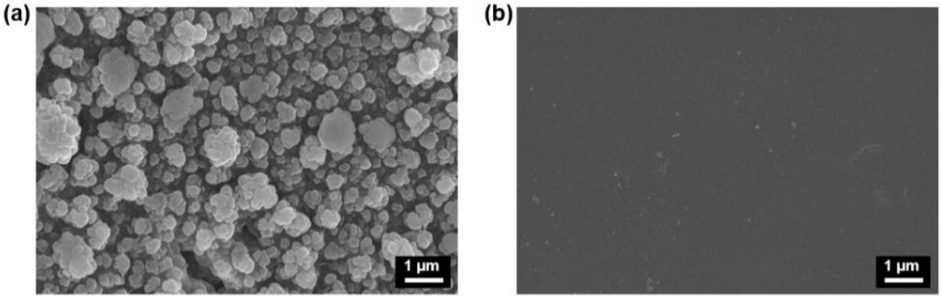

Figure S26. SEM images of the crosslinked *α*-CD powders and crosslinked *α*-CD-in-TpPa-1 mixed matrix membrane (crosslinked *α*-CD-in-TpPa-1 MMM) surface, respectively.

Preparation of crosslinked *α*-CD-in-TpPa-1 MMM was performed by the following procedure: 20 mg of *α*-CD was added to the prepared ECH solution (20 mg dissolved in 14 ml of 25% NaOH solution), which was crosslinked at 65 °C for 1 h. Then the solution was neutralized by adding 6 ml of acetic acid. Afterwards, a certain amount of this solution was added the monomer of Pa-1 as the aqueous phase. The organic phase was prepared by dissolving Tp into the solvent of toluene. Finally, the crosslinked *α*-CD-in-TpPa-1 MMM was prepared by the interfacial polymerization on the polyacrylonitrile (PAN) ultrafiltration substrate (PAN50, molecular weight cut off ~50000 g mol^{-1}). It should be noted that the *α*-CD content is kept almost the same as in LA-α-CD-in-TpPa-1 membrane.

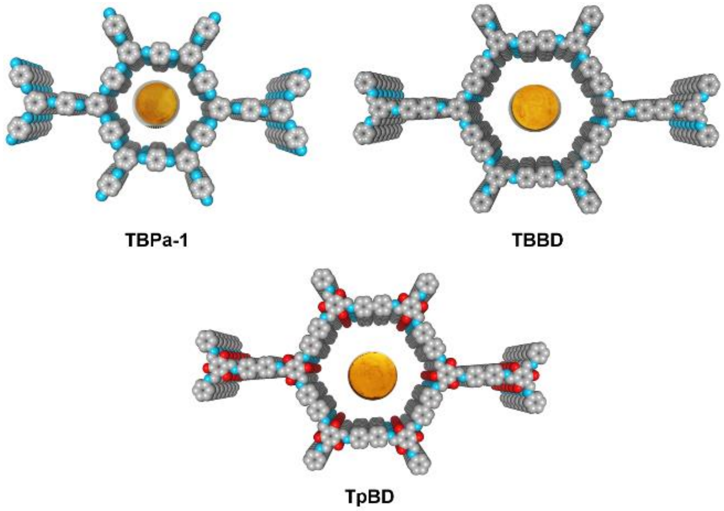

Figure S27. Schematic of the molecular structure of the COFs used for synthesis of LA-α-CD-in-COF membranes. The insert is the digital photograph of the synthesized membrane, respectively.

The reported pore size of the TBPa-1, TBBD, and TPBD is ~ 1.83 nm, ~ 2.4 nm, and ~ 2.31 nm, respectively,[S11-S13] which is large enough to accomondate the α-CD molecules.

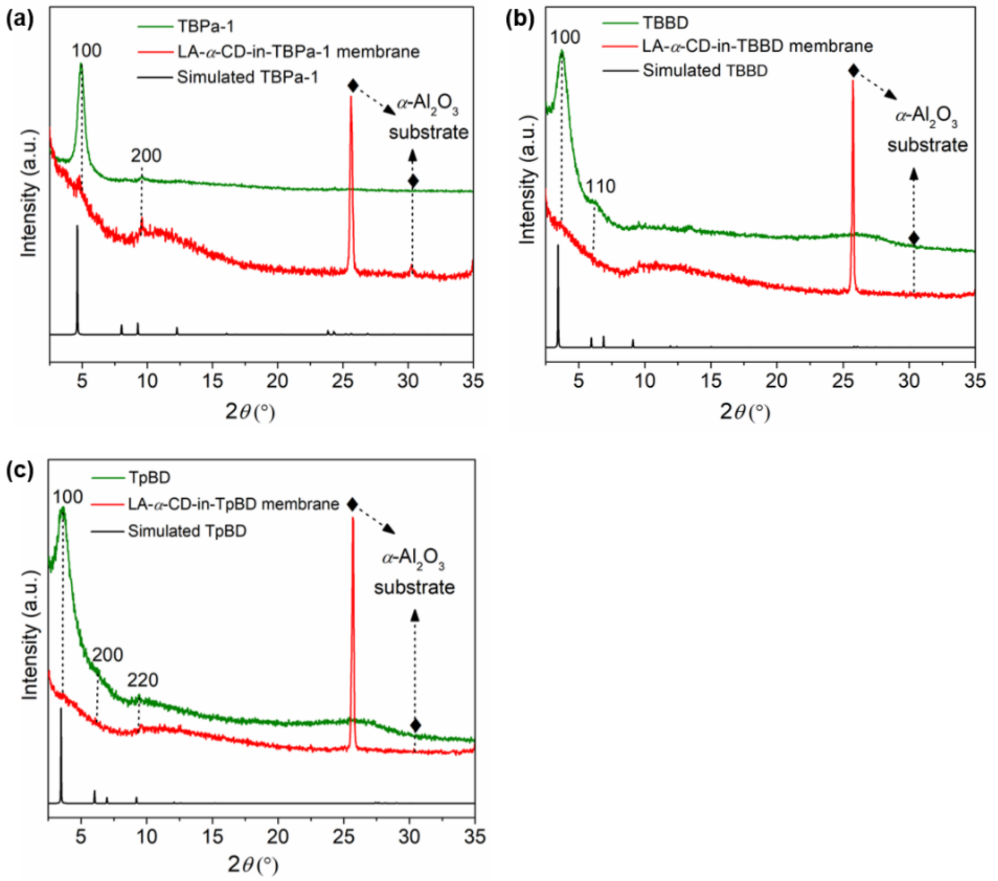

Figure S28. XRD patterns of synthesized LA-α-CD-in-COF membranes.

From Figure S19, all of the membranes have the characteristic diffraction peaks of corresponding COF, as compared to the simulated patterns. The 100 diffraction signals of the membranes are not as strong as the powder, probably owing to the thin thickness of the COF layer and a certain of oriented growth of 2D COF in its eclipsed stacking structure parallel to the surface of the α-Al_2O_3 substrate.[S14]

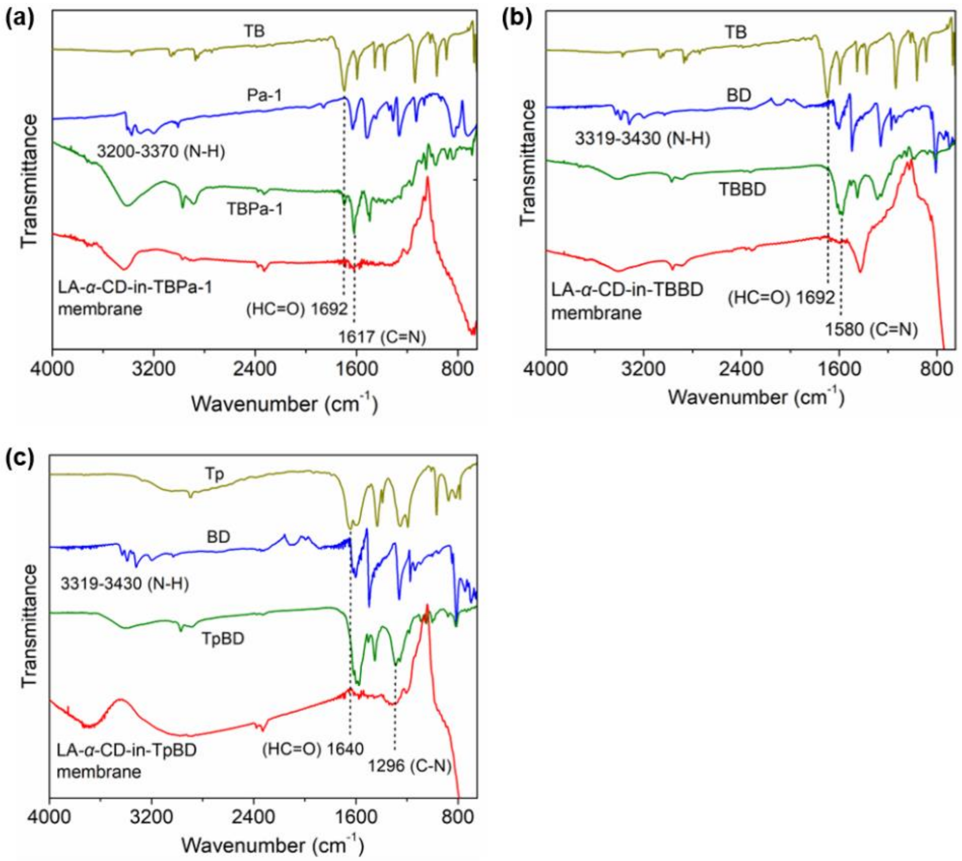

Figure S29. FTIR spectra of synthesized LA-α-CD-in-COF membranes.

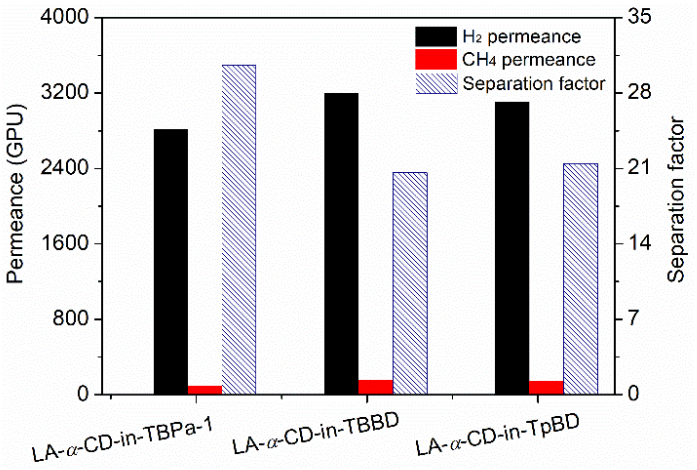

Figure S30. Permeances and separation selectivities of synthesized LA-α-CD-in-COF membranes. (298 K and 1 bar).

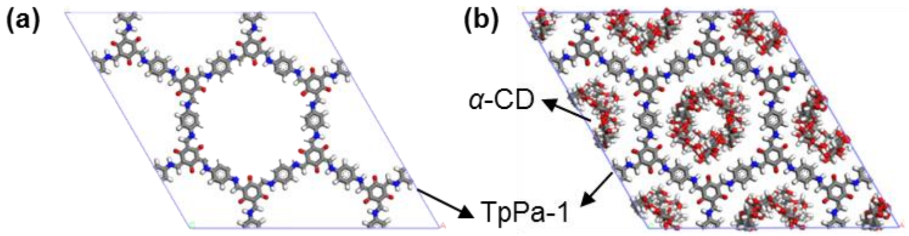

Figure S31. (a) Atomic structure of (a) TpPa-1 and (b) LA-α-CD-in-TpPa-1.

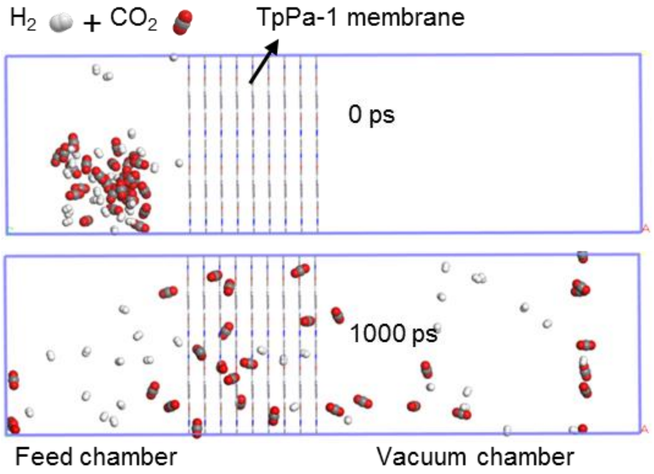

Figure S32. Simulation system with snapshot at 0, and 1000 ps for the permeation of an equimolar H_2/CO_2 mixture (30 H_2 and 30 CO_2 molecules in the feed chamber) through an TpPa-1 channel without LA-α-CD.

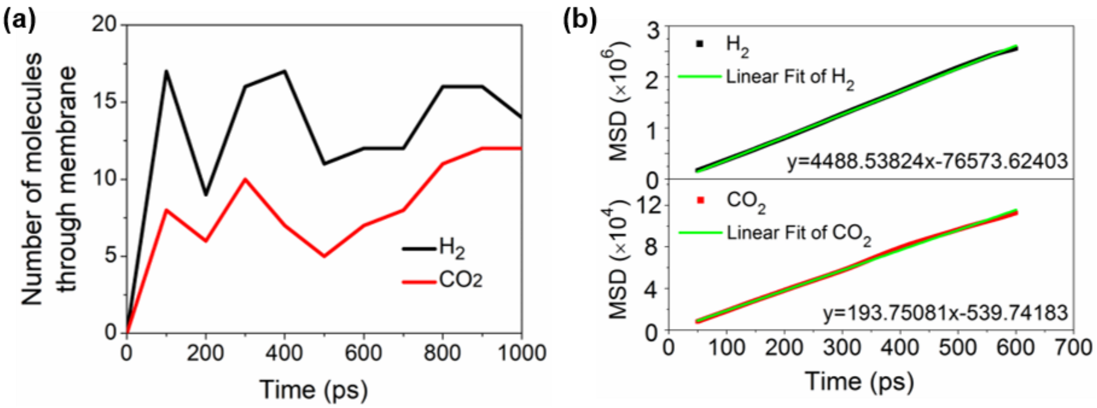

Figure S33. (a) The number of gas molecules that passed through an TpPa-1 channel without LA-α-CD in MD simulation as a function of simulation time for mixed-gas permeation. (b) The calculated mean square displacement (MSD) values versus simulation time for H_2 and CO_2.

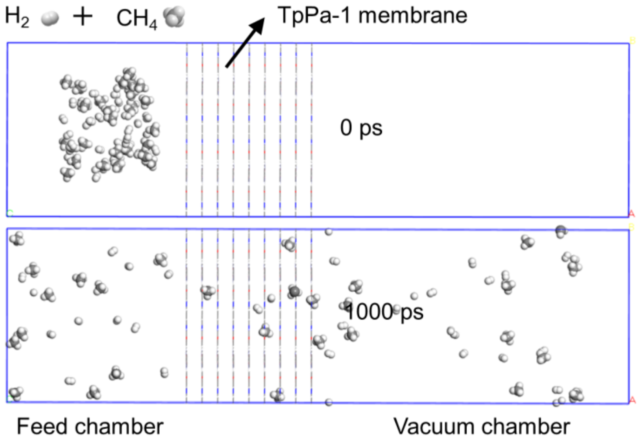

Figure S34. Simulation system with snapshot at 0, and 1000 ps for the permeation of an equimolar H_2/CH_4 mixture (30 H_2 and 30 CH_4 molecules in the feed chamber) through an TpPa-1 channel without LA-α-CD.

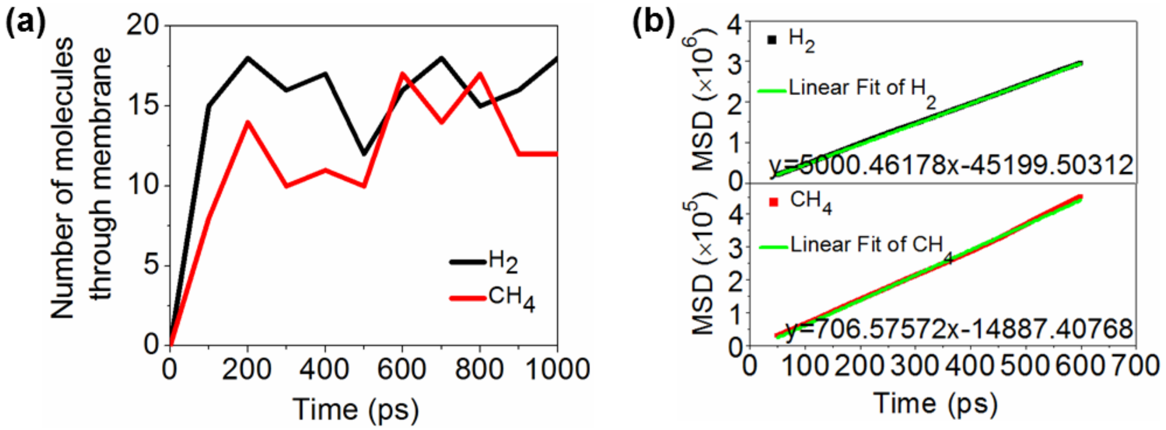

Figure S35. (a) The number of gas molecules that passed through an TpPa-1 channel without LA-α-CD in MD simulation as a function of simulation time for mixed-gas permeation. (b) The calculated mean square displacement (MSD) values versus simulation time for H_2 and CH_4.

In an TpPa-1 channel without LA-α-CD (Figure S34, Figure S35a), 60% of the H_2 molecules (18 out of 30 molecules) passed but accompanied by 40% of the CH_4 molecules (12 out of 30 molecules) within 1000 ps. The calculated H_2 diffusion coefficient is only 7.1 times larger than the CH_4 one (Figure S35b).

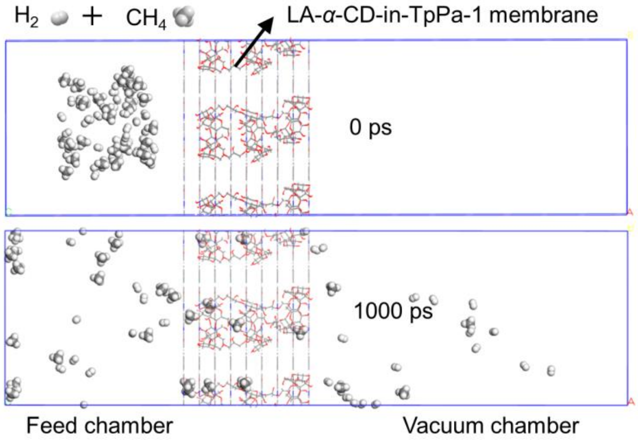

Figure S36. Simulation system with snapshot at 0, and 1000 ps for the permeation of an equimolar H_2/CH_4 mixture (30 H_2 and 30 CH_4 molecules in the feed chamber) through an LA-*α*-CD-in-TpPa-1 channel.

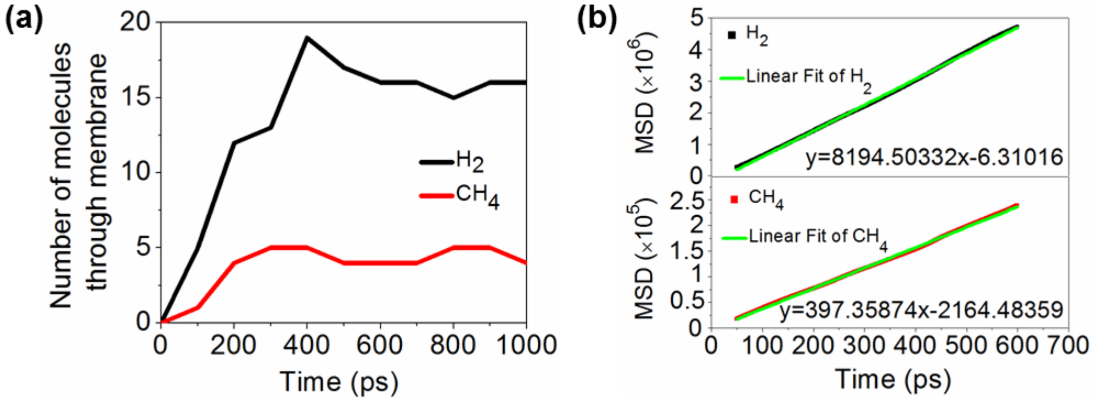

Figure S37. (a) The number of gas molecules that passed through an LA-*α*-CD-in-TpPa-1 channel without LA-*α*-CD in MD simulation as a function of simulation time for mixed-gas permeation. (b) The calculated mean square displacement (MSD) values versus simulation time for H_2 and CH_4.

It was found that the H_2 molecules also preferentially permeate through the LA-α-CD-in-TpPa-1 channels, and the permeation of CH_4 molecules was suppressed in spite of weak adsorptive interaction. As shown in Figure S36 and Figure S37a, 53.3% of the H_2 molecules (16 out of 30 molecules) in the feed chamber could pass through the LA-α-CD-in-TpPa-1 membrane to the permeate chamber within 1000 ps, accompanied by only 13.3% of the CH_4 molecules (4 out of 30 molecules), demonstrating a faster H_2 permeation. The caculated H_2 diffusion coefficient is about 20.6 times as large as the CH_4 diffusivity (Figure S37b).

Table S1 Diameter of hydrated ions and molecule size of water-soluble dyes.

Dye molecule or ion	Hydrated diameter or molecule size (nm)
Cl^-	0.664
Na^+	0.716
CO_3^{2-}	0.788
Gd^{3+}	0.918
SO_4^{2-}	1.00
Methylene blue (MEB)	1.25 nm×0.51 nm
Chrome black T (CBT)	1.55 nm×0.88 nm
Acid fuchsin (AF)	1.13 nm×1.17 nm
Rose bengal (RB)	1.2 nm×1.54 nm
Congo red (CR)	2.56 nm×0.73 nm
Methyl blue (MB)	2.36 nm×1.74 nm

Table S2 Performance comparison among various membranes towards Na_2SO_4 rejection.

Membrane type	Thickness (nm)	P (L m^{-2} h^{-1} MPa^{-1})	Salt	Rejection (%)	Ref.
PA/TpPa-1_{50}/PES	120	171.3	Na_2SO_4	>90	[S15]
PA/PDA-COF/PAN	11	207.1	Na_2SO_4	93.4	[S16]
IISERP-COOH-COF1	2700	5.0	Na_2SO_4	96.3	[S17]
TpHz/PES	500	40.5	Na_2SO_4	58.3	[S18]
PA-SNW-1/PES	300	192.5	Na_2SO_4	83.5	[S19]
LA-α-CD-in-TpPa-1	1500	67.5	Na_2SO_4	98.4	This work

Table S3 A summary of the H_2/CO_2 separation performance of various membranes.

Membrane materials		Thickness (μm)	Temperature (°C)	H_2 permeance (GPU)	Separation factor (H_2/CO_2)	Ref.
Zeolite	AIPO$_4$	6	35	990.3	9.7	[S20]
	A-type	3.8	35	294.7	9.4	[S21]
	DDR+CVD	15	550	58.9	32.7	[S22]
	LTA	10	20	2387.4	10.9	[S23]
	LTA (ITQ-29)	12	300	1178	7.8	[S24]
	LTA (triple layer)	10.5	100	471.6	12.5	[S25]
	LTA (ALPO$_4$)	25	200	560	10.9	[S26]
	MFI+CVD	2	450	442.1	8.2	[S27]
	MFI+CCD	2	500	29.5	45.6	[S28]
	NaA	5	65	1491.4	92	[S29]
	SAPO-34	26	30	26526.8	16.7	[S30]
	SAPO-34/Pd (CVD)	25	30	4362.2	20.8	[S31]
	B-ZSM-5+MDES	3.5	400	294.7	47	[S32]
	ZSM-5/Silicalite-1	10	500	353.7	23	[S33]
	ZSM-5/Silicalite-1	9.5	25	377.3	25.3	[S34]
	RUB-15	0.3	200	600	100	[S35]
MOF	HKUST-1	60	25	2947.4	6.8	[S36]
	ZIF-7	1.5	200	227.2	6.48	[S37]
	ZIF-22	40	50	489.3	7.2	[S38]
	NH_2-MIL-53 (Al)	15	15	5850.6	30.9	[S39]
	Amine-Mg-MOF-74	10	25	224	28	[S40]
	ZIF-7	2	200	27.1	8.4	[S41]
	ZIF-8	12	25	294.7	6	[S42]
	ZIF-8	2	25	1267.4	3.28	[S43]
	MIL-96 (Al)	8	RT	1532.6	8.8	[S44]
	$Zn_2(Bim)_3$	0.01	200	1918.8	128.4	[S45]
	Oriented ZIF-95	0.6	100	2360.9	32.2	[S46]
	Random ZIF-95	20	100	253.2	25.5	[S47]
	MIS-ZIF-L	~0	25	4033	321	[S48]
	Zr-Anth-MOF	1	25	55	26	[S49]
	ZIF-8@GO	20	250	379.3	14.9	[S50]
	$Zn_2(bim)_4$	--	120	2700	291	[S51]

	MAMS-1	0.04	RT	553	235	[S52]
GO/ Graphene	GO/α-alumina-hollow fiber	0.3	20	353.7	10.22	[S53]
	GO/AAO	0.02	25	1494.3	48	[S54]
	GO/ γ-alumina-Tubular	--	20	1739	38.5	[S55]
	SOD/GO-M1	~ 1	25	1050	105	[S56]
	GO-B	0.2	RT	92.8	100.1	[S57]
	GO-0.5	1	25	79.6	20.9	[S58]
	EFDA-GO	1	25	1184	29	[S59]
	GO	0.01	25	35	30	[S60]
	GO-MEM-S250	0.5	RT	167.5	26.38	[S61]
	CGO	0.05	25	51.5	21.3	[S62]
	GO30ND^{+}	--	--	3300	180	[S63]
POM	BILP-101x (400 nm)	0.4	150	24.2	39.5	[S64]
	P33DT-ThC4	2	25	308.8	50	[S65]
	TAM-DBTP-CMP@PSU	12.5	25	518.1	37.3	[S66]
	CMT	1	30	29384	6.2	[S67]
	[COF-300]-[Zn2(bdc)2(dabco)]	97	RT	1344	12.6	[S68]
	[COF-300]-[ZIF-8]	100	RT	1055.2	13.5	[S68]
	COF-300	40	RT	2688	6.0	[S68]
	COF-LZU1	0.5	25	3684.3	6.0	[S69]
	ACOF-1	0.6	25	2019	14.1	[S69]
	COF-LZU1–ACOF-1	1	25	660.2	24.2	[S69]
	CTF-1	0.1	RT	5010.6	17.4	[S70]
	TpPa-1-30/GO-10	0.3	25	3144.9	25.57	[S71]
	[COF-300]-[UiO-66]	100	RT	1173.1	17.2	[S72]
	TpEBr@TpPa-SO_3Na	0.041	150	2566	22.6	[S73]
	Vertically aligned TFB-BD	2	25	3802.2	25.6	[S74]
	TpTG$_{Cl}$@TpPa-SO_3H/COF-LZU1	0.155	150	2163	26	[S75]
LA-α-CD-in-TpPa-1		1.5	25	3023.3	34.9	This work

POM: Porous organic materials; MOFs: Metal-organic frameworks; PIMs: Polymers of intrinsic microporosity; TR polymer: Thermally rearranged polymer; PBI: Polybenzimidazole; POFs: Porous organic frameworks. CMT: Conjugated microporous thermoset.

Table S4 A summary of the H_2/CH_4 separation performance of various membranes.

Membrane materials		Thickness (μm)	Temperature (°C)	H_2 permance (GPU)	Separation factor (H_2/CH_4)	Ref.
Zeolite	AIPO-18	4-10	25	265.3	18.7	[S76]
	AIPO-18	8	RT	300	22	[S77]
	FAU	2.7	50	551.2	9.9	[S78]
	FAU	2.5	100	1179	4	[S79]
	Ag-LTA	4.5	50	677.9	21.3	[S80]
	SAPO-34	15	25	70.7	25	[S81]
	SAPO-34	10	200	159.2	7.9	[S82]
	SAPO-34	4	25	20514	14.7	[S83]
	SAPO-34/Carbon	2	25	294.7	97	[S84]
	SSZ-13	4-6	200	533.4	16	[S85]
	SSZ-13	4-6	RT	630	22	[S86]
	SSZ-13+CLD	5-6	25	1040.4	5.9	[S87]
	Zeolite L/Carbon	3-4	25	294.7	62.6	[S88]
	Zeolite L/Carbon	3-4	25	26.5	103	[S89]
	DDR	2-3	25	270	75	[S90]
	CHA	4.5-6	40	2690	26.5	[S91]
MOF	HKUST-1	3	RT	5924.3	5.4	[S92]
	HKUST-1	20	RT	884.2	8.8	[S93]
	ZIF-7-8	2	25	250.5	11.4	[S94]
	ZIF-8	0.5	25	24463.6	16.2	[S95]
	ZIF-8	1	RT	59243.1	9.1	[S96]
	ZIF-8	2	RT	1797.9	10.2	[S97]
	ZIF-9	30	25	1606.3	10.35	[S98]
	ZIF-90	20	200	571.8	18.9	[S99]
	ZIF-90	20	225	854.7	70.5	[S100]
	ZIF-94	7	35	47.2	85.6	[S101]
	ZIF-8	35	35	589.5	10.5	[S102]
	ZIF-8	30	25	178	11.2	[S103]
	ZIF-8	20	150	648.4	31.5	[S104]
	ZIF-8	2	RT	4539	13	[S105]
	ZIF-8	6	RT	468.6	11.4	[S106]
	ZIF-8	20	RT	433.3	12.5	[S107]

	ZIF-90	20	225	840	70.5	[S108]
	2abIm-VPLT-RTD-ZIF-8	3	25	442.1	140	[S109]
	Oriented ZIF-95	0.6	100	2328.5	53.7	[S46]
	Random ZIF-95	0.6	100	501.1	40.3	[S47]
	ZIF-62 glass	70	RT	56	50	[S110]
	ZIF-67	3	25	460	41.9	[S111]
	JCU-150	30-40	RT	530	26.3	[S112]
GO/ Graphene	GO/α-alumina-hollow fiber	0.3	20	365.5	4.67	[S53]
	Ag/IL-GO (18%)	2.4	RT	470	40	[S113]
	SOD/GO-M1	~ 1	25	1200	80	[S56]
	SOD/GO-M2	~ 1	25	3900	4.9	[S56]
	GO-B	0.2	RT	139.4	27.71	[S57]
	GO-MEM-S250	0.5	RT	167.5	12.52	[S61]
	Graphene	0.08	150	3400	25.1	[S114]
	GO-thiourea	~ 0.35	25	589	66.7	[S115]
	Single-layer graphene	--	150	1326	17	[S116]
	Single-layer graphene	--	150	619	20	[S37]
	Single-layer graphene	--	25	~7200	37.5	[S117]
	Porous single-layer graphene	--	20	12020	240.4	[S118]
		--	100	15950	129.7	
		--	150	18580	106.8	
	Double-layer graphene perforated by ion irradiation and O_2 etching	--	--	3400	9.2	[S119]
POM	CC3	0.05	RT	4509.5	16.5	[S120]
	P33DT-ThC4	2	25	302.9	73.6	[S65]
	TAM-DBTP-CMP@PSU	12.5	25	518.1	30.8	[S66]
	CMT	1	30	29384	10.8	[S67]
	COF-320	4	RT	1671.2	2.5	[S121]
	COF-LZU1	0.5	25	3389.5	9.65	[S69]
	ACOF-1	0.6	25	1744.8	24.67	[S69]
	COF-LZU1–ACOF-1	1	25	536.4	100.2	[S69]
	Vertically aligned TFB-BD	2	25	3477.9	24.9	[S74]
LA-α-CD-in-TpPa-1		1.5	25	2995	38.1	This work

References

[S1] Uyar, T.; Hunt, M. A.; Gracz, H. S.; Tonelli, A. E. Crystalline Cyclodextrin Inclusion Compounds Formed with Aromatic Guests: Guest-Dependent Stoichiometries and Hydration-Sensitive Crystal Structures. *Cryst. Growth Des.* **2006**, *6*, 1113-1119.

[S2] Kayaci, F.; Uyar, T. Solid Inclusion Complexes of Vanillin with Cyclodextrins: Their Formation, Characterization, and High-Temperature Stability. *J. Agr. Food Chem.* **2011**, *59*, 11772-11778.

[S3] Harada, A.; Okada, M.; Li, J.; Kamachi, M. Preparation and Characterization of Inclusion Complexes of Poly(propylene glycol) with Cyclodextrins. *Macromolecules* **1995**, *28*, 8406-8411.

[S4] Saenger, W.; Jacob, J.; Gessler, K.; Steiner, T.; Hoffmann, D.; Sanbe, H.; Koizumi, K.; Smith, S. M.; Takaha, T. Structures of the Common Cyclodextrins and Their Larger Analogues Beyond the Doughnut. *Chem. Rev.* **1998**, *98*, 1787-1802.

[S5] Harada, A.; Kamachi, M. Complex formation between poly (ethylene glycol) and *α*-cyclodextrin. *Macromolecules* 1990, 23, 2821-2823.

[S6] Harata, K. Structural Aspects of Stereodifferentiation in the Solid State. *Chem. Rev.* **1998**, *98*, 1803-1828.

[S7] He, Y.; Fu, P.; Shen, X.; Gao, H. Cyclodextrin-based aggregates and characterization by microscopy. *Micron* **2008**, *39*, 495-516.

[S8] Rusa, C. C.; Bullions, T. A.; Fox, J.; Porbeni, F. E.; Wang, X.; Tonelli, A. E. Inclusion Compound Formation with a New Columnar Cyclodextrin Host. *Langmuir* **2002**, *18*, 10016-10023.

[S9] Jensen, F. Activation energies and the arrhenius equation. *Qual. Reliab. Eng. Int.* **1985**, *1*, 13-17.

[S10] Liang, Y.; Teng, X.; Chen, R.; Zhu, Y.; Jin, J.; Lin, S. Polyamide nanofiltration membranes from emulsion-mediated interfacial polymerization. *ACS EST Eng.* **2021**, *1*, 533–542.

[S11] Ding, S.-Y.; Gao, J.; Wang, Q.; Zhang, Y.; Song, W.-G.; Su, C.-Y.; Wang, W. Construction of Covalent Organic Framework for Catalysis: Pd/COF-LZU1 in Suzuki–Miyaura Coupling Reaction. *J. Am. Chem. Soc.* **2011**, *133*, 19816-19822.

[S12] Vitaku, E.; Dichtel, W. R. Synthesis of 2D Imine-Linked Covalent Organic Frameworks through Formal Transimination Reactions. *J. Am. Chem. Soc.* **2017**, *139*, 12911-12914.

[S13] Biswal, B. P.; Chandra, S.; Kandambeth, S.; Lukose, B.; Heine, T.; Banerjee, R. Mechanochemical Synthesis of Chemically Stable Isoreticular Covalent Organic Frameworks. *J. Am. Chem. Soc.* **2013**, *135*, 5328-5331.

[S14] Gou, X.; Zhang, Q.; Wu, Y.; Zhao, Y.; Shi, X.; Fan, X.; Huang, L.; Lu, G. Preparation and engineering of oriented 2D covalent organic framework thin films. *RSC Adv.* **2016**, *6*, 39198-39203.

[S15] Wang, M.; Guo, W.; Jiang, Z.; Pan, F. Reducing active layer thickness of polyamide composite membranes using a covalent organic framework interlayer in interfacial polymerization. *Chin. J. Chem. Eng.* **2020**, *28*, 1039-1045.

[S16] Wu, M.; Yuan, J.; Wu, H.; Su, Y.; Yang, H.; You, X.; Zhang, R.; He, X.; Khan, N. A.; Kasher, R.; et al. Ultrathin nanofiltration membrane with polydopamine-covalent organic framework interlayer for enhanced permeability and structural stability. *J. Membr. Sci.* **2019**, *576*, 131-141.

[S17] Liu, C.; Jiang, Y.; Nalaparaju, A.; Jiang, J.; Huang, A. Post-synthesis of a covalent organic framework nanofiltration membrane for highly efficient water treatment. *J. Mater. Chem. A* **2019**, *7*, 24205-24210.

[S18] Wang, R.; Wei, M.; Wang, Y. Secondary growth of covalent organic frameworks (COFs) on porous substrates for fast desalination. *J. Membr. Sci.* **2020**, *604*, 118090.

[S19] Wang, C.; Li, Z.; Chen, J.; Li, Z.; Yin, Y.; Cao, L.; Zhong, Y.; Wu, H. Covalent organic framework modified polyamide nanofiltration membrane with enhanced performance for desalination. *J. Membr. Sci.* **2017**, *523*, 273-281.

[S20] Guan, G.; Tanaka, T.; Kusakabe, K.; Sotowa, K.-I.; Morooka, S. Characterization of AlPO4-type molecular sieving membranes formed on a porous *α*-alumina tube. *J. Membr. Sci.* **2003**, *214*, 191-198.

[S21] Aoki, K.; Kusakabe, K.; Morooka, S. Gas permeation properties of A-type zeolite membrane formed on porous substrate by hydrothermal synthesis. *J. Membr. Sci.* **1998**, *141*, 197-205.

[S22] Zheng, Z.; Hall, A. S.; Guliants, V. V. Synthesis, characterization and modification of DDR membranes grown on α-alumina supports. *J. Mater. Sci.* **2008**, *43*, 2499-2502.

[S23] Li, X.; Li, K.; Tao, S.; Ma, H.; Xu, R.; Wang, B.; Wang, P.; Tian, Z. Ionothermal synthesis of LTA-type aluminophosphate molecular sieve membranes with gas separation performance. *Micropor. Mesopor. Mater.* **2016**, *228*, 45-53.

[S24] Huang, A.; Caro, J. Steam-stable hydrophobic ITQ-29 molecular sieve membrane with H2 selectivity prepared by secondary growth using Kryptofix 222 as SDA. *Chem. Commun.* **2010**, *46*, 7748-7750.

[S25] Huang, A.; Wang, N.; Caro, J. Synthesis of multi-layer zeolite LTA membranes with enhanced gas separation performance by using 3-aminopropyltriethoxysilane as interlayer. *Micropor. Mesopor. Mater.* **2012**, *164*, 294-301.

[S26] Huang, A.; Caro, J. Highly oriented, neutral and cation-free AlPO4 LTA: from a seed crystal monolayer to a molecular sieve membrane. *Chem. Commun.* **2011**, *47*, 4201-4203.

[S27] Zhu, X.; Wang, H.; Lin, Y. S. Effect of the Membrane Quality on Gas Permeation and Chemical Vapor Deposition Modification of MFI-Type Zeolite Membranes. *Ind. Eng. Chem. Res.* **2010**, *49*, 10026-10033.

[S28] Hong, Z.; Sun, F.; Chen, D.; Zhang, C.; Gu, X.; Xu, N. Improvement of hydrogen-separating performance by on-stream catalytic cracking of silane over hollow fiber MFI zeolite membrane. *Int. J. Hydrogen Energy* **2013**, *38*, 8409-8414.

[S29] Dey, K. P.; Kundu, D.; Chatterjee, M.; Naskar, M. K. Preparation of NaA Zeolite Membranes Using Poly(Ethyleneimine) as Buffer Layer, and Study of Their Permeation Behavior. *J. Am. Ceram. Soc.* **2013**, *96*, 68-72.

[S30] Das, J. K.; Das, N.; Bandyopadhyay, S. Highly oriented improved SAPO 34 membrane on low cost support for hydrogen gas separation. *J. Mater. Chem. A* **2013**, *1*, 4966-4973.

[S31] Das, J. K.; Das, N. Mercaptoundecanoic Acid Capped Palladium Nanoparticles in a SAPO 34 Membrane: A Solution for Enhancement of H_2/CO_2 Separation Efficiency. *ACS Appl. Mater. Interfaces* **2014**, *6*, 20717-20728.

[S32] Hong, M.; Falconer, J. L.; Noble, R. D. Modification of Zeolite Membranes for H_2 Separation by Catalytic Cracking of Methyldiethoxysilane. *Ind. Eng. Chem. Res.* **2005**, *44*, 4035-4041.

[S33] Wang, H.; Dong, X.; Lin, Y. S. Highly stable bilayer MFI zeolite membranes for high temperature hydrogen separation. *J. Membr. Sci.* **2014**, *450*, 425-432.

[S34] Wang, H.; Lin, Y. S. Synthesis and modification of ZSM-5/silicalite bilayer membrane with improved hydrogen separation performance. *J. Membr. Sci.* **2012**, *396*, 128-137.

[S35] Dakhchoune, M.; Villalobos, L. F.; Semino, R.; Liu, L.; Rezaei, M.; Schouwink, P.; Avalos, C. E.; Baade, P.; Wood, V.; Han, Y.; Ceriotti, M.; Agrawal, K. V. Gas-sieving zeolitic membranes fabricated by condensation of precursor nanosheets. *Nat. Mater.* **2021**, *20*, 362-369.

[S36] Guo, H.; Zhu, G.; Hewitt, I. J.; Qiu, S. "Twin Copper Source" Growth of Metal−Organic Framework Membrane: $Cu_3(BTC)_2$ with High Permeability and Selectivity for Recycling H_2. *J. Am. Chem. Soc.* **2009**, *131*, 1646-1647.

[S37] Li, Y. S.; Liang, F. Y.; Bux, H.; Feldhoff, A.; Yang, W. S.; Caro, J. Molecular sieve membrane: supported metal-organic framework with high hydrogen selectivity. *Angew. Chem., Int. Ed.* **2010**, *122, 558-561*.

[S38] Huang, A.; Bux, H.; Steinbach, F.; Caro, J. Molecular-sieve membrane with hydrogen permselectivity: ZIF-22 in LTA topology prepared with 3-aminopropyltriethoxysilane as covalent linker. *Angew. Chem., Int. Ed.* **2010**, *49*, 4958-4961.

[S39] Zhang, F.; Zou, X.; Gao, X.; Fan, S.; Sun, F.; Ren, H.; Zhu, G. Hydrogen Selective NH_2-MIL-53(Al) MOF Membranes with High Permeability. *Adv. Funct. Mater.* **2012**, *22*, 3583-3590.

[S40] Wang, N.; Mundstock, A.; Liu, Y.; Huang, A.; Caro, J. Amine-modified Mg-MOF-74/CPO-27-Mg membrane with enhanced H_2/CO_2 separation. *Chem. Eng. Sci* **2015**, *124*, 27-36.

[S41] Li, Y.-S.; Bux, H.; Feldhoff, A.; Li, G.-L.; Yang, W.-S.; Caro, J. Controllable Synthesis of Metal–Organic Frameworks: From MOF Nanorods to Oriented MOF Membranes. *Adv. Mater.* **2010**, *22*, 3322-3326.

[S42] Bux, H.; Feldhoff, A.; Cravillon, J.; Wiebcke, M.; Li, Y.-S.; Caro, J. Oriented Zeolitic Imidazolate Framework-8 Membrane with Sharp H_2/C_3H_8 Molecular Sieve Separation. *Chem. Mater.* **2011**, *23*, 2262-2269.

[S43] Huang, K.; Dong, Z.; Li, Q.; Jin, W. Growth of a ZIF-8 membrane on the inner-surface of a ceramic hollow fiber via cycling precursors. *Chem. Commun.* **2013**, *49*, 10326-10328.

[S44] Knebel, A.; Friebe, S.; Bigall, N. C.; Benzaqui, M.; Serre, C.; Caro, J. Comparative Study of MIL-96(Al) as Continuous Metal–Organic Frameworks Layer and Mixed-Matrix Membrane. *ACS Appl. Mater. Interfaces* **2016**, *8*, 7536-7544.

[S45] Peng, Y.; Li, Y.; Ban, Y.; Yang, W. Two-Dimensional Metal-Organic Framework Nanosheets for Membrane-Based Gas Separation. *Angew. Chem., Int. Ed.* **2017**, *56*, 9757-9761.

[S46] Ma, X.; Wan, Z.; Li, Y.; He, X.; Caro, J.; Huang, A. Anisotropic Gas Separation in Oriented ZIF-95 Membranes Prepared by Vapor-Assisted In-Plane Epitaxial Growth. *Angew. Chem., Int. Ed.* **2020**, *59*, 20858-20862.

[S47] Ma, X.; Li, Y.; Huang, A. Synthesis of nano-sheets seeds for secondary growth of highly hydrogen permselective ZIF-95 membranes. *J. Membr. Sci.* **2020**, *597*, 117629.

[S48] Yang, K.; Hu, S.; Ban, Y.; Zhou, Y.; Cao, N.; Zhao, M.; Xiao, Y.; Li, W.; Yang, W. ZIF-L membrane with a membrane-interlocked-support composite architecture for H_2/CO_2 separation. *Sci. Bull.* **2021**, *66*, 1869-1876.

[S49] Ghalei, B.; Wakimoto, K.; Wu, C. Y.; Isfahani, A. P.; Yamamoto, T.; Sakurai, K.; Higuchi, M.; Chang, B. K.; Kitagawa, S.; Sivaniah, E. Rational Tuning of Zirconium Metal–Organic Framework Membranes for Hydrogen Purification. *Angew. Chem., Int. Ed.* **2019**, *58*, 19034-19040.

[S50] Huang, A.; Liu, Q.; Wang, N.; Zhu, Y.; Caro, J. Bicontinuous Zeolitic Imidazolate Framework ZIF-8@GO Membrane with Enhanced Hydrogen Selectivity. *J. Am. Chem. Soc.* **2014**, *136*, 14686-14689.

[S51] Peng, Y.; Li, Y.; Ban, Y.; Jin, H.; Jiao, W.; Liu, X.; Yang, W. Metal-organic framework nanosheets as building blocks for molecular sieving membranes. *Science* **2014**, *346*, 1356-1359.

[S52] Wang, X.; Chi, C.; Zhang, K.; Qian, Y.; Gupta, K. M.; Kang, Z.; Jiang, J.; Zhao, D. Reversed thermo-switchable molecular sieving membranes composed of two-dimensional metal-organic nanosheets for gas separation. *Nat. Commun.* **2017**, *8*, 14460.

[S53] Huang, K.; Yuan, J.; Shen, G.; Liu, G.; Jin, W. Graphene oxide membranes supported on the ceramic hollow fibre for efficient H_2 recovery. *Chinese J. Chem. Eng.* **2017**, *25*, 752-759.

[S54] Chi, C.; Wang, X.; Peng, Y.; Qian, Y.; Hu, Z.; Dong, J.; Zhao, D. Facile Preparation of Graphene Oxide Membranes for Gas Separation. *Chem. Mater.* **2016**, *28*, 2921-2927.

[S55] Zeynali, R.; Ghasemzadeh, K.; Sarand, A. B.; Kheiri, F.; Basile, A. Performance evaluation of graphene oxide (GO) nanocomposite membrane for hydrogen separation: Effect of dip coating sol concentration. *Sep. Purif. Technol.* **2018**, *200*, 169-176.

[S56] Guo, H.; Kong, G.; Yang, G.; Pang, J.; Kang, Z.; Feng, S.; Zhao, L.; Fan, L.; Zhu, L.; Vicente, A.; Peng, P.; Yan, Z.; Sun, D.; Mintova, S. Cross-Linking between Sodalite Nanoparticles and Graphene Oxide in Composite Membranes to Trigger High Gas Permeance, Selectivity, and Stability in Hydrogen Separation. *Angew. Chem., Int. Ed.* **2020**, *59*, 6284-6288.

[S57] Ibrahim, A. F. M.; Banihashemi, F.; Lin, Y. S. Graphene oxide membranes with narrow inter-sheet galleries for enhanced hydrogen separation. *Chem. Commun.* **2019**, *55*, 3077-3080.

[S58] Guan, K.; Shen, J.; Liu, G.; Zhao, J.; Zhou, H.; Jin, W. Spray-evaporation assembled graphene oxide membranes for selective hydrogen transport. *Sep. Purif. Technol.* **2017**, *174*, 126-135.

[S59] Shen, J.; Liu, G.; Huang, K.; Chu, Z.; Jin, W.; Xu, N. Subnanometer Two-Dimensional Graphene Oxide Channels for Ultrafast Gas Sieving. *ACS Nano* **2016**, *10*, 3398-3409.

[S60] Kim, H. W.; Yoon, H. W.; Yoon, S.-M.; Yoo, B. M.; Ahn, B. K.; Cho, Y. H.; Shin, H. J.; Yang, H.; Paik, U.; Kwon, S.; Choi, J.-Y.; Park, H.B. Selective Gas Transport Through Few-Layered Graphene and Graphene Oxide Membranes. *Science* **2013**, *342*, 91-95.

[S61] Ibrahim, A. F. M.; Lin, Y. S. Synthesis of graphene oxide membranes on polyester substrate by spray coating for gas separation. *Chem. Eng. Sci* **2018**, *190*, 312-319.

[S62] Cheng, L.; Guan, K.; Liu, G.; Jin, W. Cysteamine-crosslinked graphene oxide membrane with enhanced hydrogen separation property. *J. Membr. Sci.* **2020**, *595*, 117568.

[S63] Huang, G.; Ghalei, B.; Pournaghshband Isfahani, A.; Karahan, H. E.; Terada, D.; Qin, D.; Li, C.; Tsujimoto, M.; Yamaguchi, D.; Sugimoto, K.; Igarashi, R.; Chang, B. K.; Li, T.; Shirakawa, M.; Sivaniah, E. Overcoming humidity-induced swelling of graphene oxide-based hydrogen membranes using charge-compensating nanodiamonds. *Nat. Energy* **2021**, *6*, 1176-1187.

[S64] Shan, M.; Liu, X.; Wang, X.; Yarulina, I.; Seoane, B.; Kapteijn, F.; Gascon, J. Facile manufacture of porous organic framework membranes for precombustion CO_2 capture. *Sci. Adv.* **2018**, *4*, eaau1698.

[S65] Zhang, M.; Jing, X.; Zhao, S.; Shao, P.; Zhang, Y.; Yuan, S.; Li, Y.; Gu, C.; Wang, X.; Ye, Y.; Feng, X.; Wang, B. Electropolymerization of Molecular-Sieving Polythiophene Membranes for H_2 Separation. *Angew. Chem., Int. Ed.* **2019**, *58*, 8768-8772.

[S66] Shao, P.; Yao, R.; Li, G.; Zhang, M.; Yuan, S.; Wang, X.; Zhu, Y.; Zhang, X.; Zhang, L.; Feng, X.; Wang, B. Molecular-Sieving Membrane by Partitioning the Channels in Ultrafiltration Membrane by In Situ Polymerization. *Angew. Chem., Int. Ed.* **2020**, *59*, 4401-4405.

[S67] Liu, W.; Jiang, S.-D.; Yan, Y.; Wang, W.; Li, J.; Leng, K.; Japip, S.; Liu, J.; Xu, H.; Liu, Y.; Park, I.; Bao, Y.; Yu, W.; Guiver, M. D.; Zhang, S.; Loh, K.P. A solution-processable and

ultra-permeable conjugated microporous thermoset for selective hydrogen separation. *Nat. Commun.* **2020**, *11*, 1633.

[S68] Fu, J.; Das, S.; Xing, G.; Ben, T.; Valtchev, V.; Qiu, S. Fabrication of COF-MOF Composite Membranes and Their Highly Selective Separation of H_2/CO_2. *J. Am. Chem. Soc.* **2016**, *138*, 7673-7680.

[S69] Fan, H.; Mundstock, A.; Feldhoff, A.; Knebel, A.; Gu, J.; Meng, H.; Caro, J. Covalent Organic Framework–Covalent Organic Framework Bilayer Membranes for Highly Selective Gas Separation. *J. Am. Chem. Soc.* **2018**, *140*, 10094-10098.

[S70] Ying, Y.; Liu, D.; Ma, J.; Tong, M.; Zhang, W.; Huang, H.; Yang, Q.; Zhong, C. A GO-assisted method for the preparation of ultrathin covalent organic framework membranes for gas separation. *J. Mater. Chem. A* **2016**, *4*, 13444-13449.

[S71] Tang, Y.; Feng, S.; Fan, L.; Pang, J.; Fan, W.; Kong, G.; Kang, Z.; Sun, D. Covalent organic frameworks combined with graphene oxide to fabricate membranes for H_2/CO_2 separation. *Sep. Purif. Technol.* **2019**, *223*, 10-16.

[S72] Das, S.; Ben, T. A [COF-300]-[UiO-66] composite membrane with remarkably high permeability and H_2/CO_2 separation selectivity. *Dalton T.* **2018**, *47*, 7206-7212.

[S73] Ying, Y.; Tong, M.; Ning, S.; Ravi, S. K.; Peh, S. B.; Tan, S. C.; Pennycook, S. J.; Zhao, D. Ultrathin Two-Dimensional Membranes Assembled by Ionic Covalent Organic Nanosheets with Reduced Apertures for Gas Separation. *J. Am. Chem. Soc.* **2020**, *142*, 4472-4480.

[S74] Fan, H.; Peng, M.; Strauss, I.; Mundstock, A.; Meng, H.; Caro, J. High-Flux Vertically Aligned 2D Covalent Organic Framework Membrane with Enhanced Hydrogen Separation. *J. Am. Chem. Soc.* **2020**, *142*, 6872-6877.

[S75] Ying, Y.; Peh, S. B.; Yang, H.; Yang, Z.; Zhao, D. Ultrathin Covalent Organic Framework Membranes via a Multi-Interfacial Engineering Strategy for Gas Separation. *Adv. Mater.* **2022**, *34*, 2104946.

[S76] Wu, T.; Wang, B.; Lu, Z.; Zhou, R.; Chen, X. Alumina-supported AlPO-18 membranes for CO_2/CH_4 separation. *J. Membr. Sci.* **2014**, *471*, 338-346.

[S77] Wang, B.; Hu, N.; Wang, H.; Zheng, Y.; Zhou, R. Improved AlPO-18 membranes for light gas separation. *J. Mater. Chem. A* **2015**, *3*, 12205-12212.

[S78] Zhou, C.; Yuan, C.; Zhu, Y.; Caro, J.; Huang, A. Facile synthesis of zeolite FAU molecular sieve membranes on bio-adhesive polydopamine modified Al_2O_3 tubes. *J. Membr. Sci.* **2015**, *494*, 174-181.

[S79] Huang, A.; Wang, N.; Caro, J. Seeding-free synthesis of dense zeolite FAU membranes on 3-aminopropyltriethoxysilane-functionalized alumina supports. *J. Membr. Sci.* **2012**, *389*, 272-279.

[S80] Xu, K.; Yuan, C.; Caro, J.; Huang, A. Silver-exchanged zeolite LTA molecular sieving membranes with enhanced hydrogen selectivity. *J. Membr. Sci.* **2016**, *511*, 1-8.

[S81] Poshusta, J. C.; Tuan, V. A.; Falconer, J. L.; Noble, R. D. Synthesis and Permeation Properties of SAPO-34 Tubular Membranes. *Ind. Eng. Chem. Res.* **1998**, *37*, 3924-3929.

[S82] Poshusta, J. C.; Tuan, V. A.; Pape, E. A.; Noble, R. D.; Falconer, J. L. Separation of light gas mixtures using SAPO-34 membranes. *AIChE J.* **2000**, *46*, 779-789.

[S83] Zhou, L.; Yang, J.; Li, G.; Wang, J.; Zhang, Y.; Lu, J.; Yin, D. Highly H_2 permeable SAPO-34 membranes by steam-assisted conversion seeding. *Int. J. Hydrogen Energy* **2014**, *39*, 14949-14954.

[S84] Li, G.; Yang, J.; Wang, J.; Xiao, W.; Zhou, L.; Zhang, Y.; Lu, J.; Yin, D. Thin carbon/SAPO-34 microporous composite membranes for gas separation. *J. Membr. Sci.* **2011**, *374*, 83-92.

[S85] Kosinov, N.; Auffret, C.; Gücüyener, C.; Szyja, B. M.; Gascon, J.; Kapteijn, F.; Hensen, E. J. M. High flux high-silica SSZ-13 membrane for CO_2 separation. *J. Mater. Chem. A* **2014**, *2*, 13083-13092.

[S86] Kosinov, N.; Auffret, C.; Sripathi, V. G. P.; Gücüyener, C.; Gascon, J.; Kapteijn, F.; Hensen, E. J. M. Influence of support morphology on the detemplation and permeation of ZSM-5 and SSZ-13 zeolite membranes. *Micropor. Mesopor. Mater.* **2014**, *197*, 268-277.

[S87] Zhou, R.; Wang, H.; Wang, B.; Chen, X.; Li, S.; Yu, M. Defect-Patching of Zeolite Membranes by Surface Modification Using Siloxane Polymers for CO_2 Separation. *Ind. Eng. Chem. Res.* **2015**, *54*, 7516-7523.

[S88] Yin, X.; Wang, J.; Chu, N.; Yang, J.; Lu, J.; Zhang, Y.; Yin, D. Zeolite L/carbon nanocomposite membranes on the porous alumina tubes and their gas separation properties. *J. Membr. Sci.* **2010**, *348*, 181-189.

[S89] Yin, X.; Chu, N.; Yang, J.; Wang, J.; Li, Z. Thin zeolite T/carbon composite membranes supported on the porous alumina tubes for CO_2 separation. *Int. J. Greenh. Gas Con.* **2013**, *15*, 55-64.

[S90] Himeno, S.; Tomita, T.; Suzuki, K.; Nakayama, K.; Yajima, K.; Yoshida, S. Synthesis and Permeation Properties of a DDR-Type Zeolite Membrane for Separation of CO_2/CH_4 Gaseous Mixtures. *Ind. Eng. Chem. Res.* **2007**, *46*, 6989-6997.

[S91] Kida, K.; Maeta, Y.; Yogo, K. Pure silica CHA-type zeolite membranes for dry and humidified CO_2/CH_4 mixtures separation. *Sep. Purif. Technol.* **2018**, *197*, 116-121.

[S92] Mao, Y.; Li, J.; Cao, W.; Ying, Y.; Sun, L.; Peng, X. Pressure-Assisted Synthesis of HKUST-1 Thin Film on Polymer Hollow Fiber at Room Temperature toward Gas Separation. *ACS Appl. Mater. Interfaces* **2014**, *6*, 4473-4479.

[S93] Shah, M. N.; Gonzalez, M. A.; McCarthy, M. C.; Jeong, H.-K. An Unconventional Rapid Synthesis of High Performance Metal–Organic Framework Membranes. *Langmuir* **2013**, *29*, 7896-7902.

[S94] Hillman, F.; Brito, J.; Jeong, H.-K. Rapid One-Pot Microwave Synthesis of Mixed-Linker Hybrid Zeolitic-Imidazolate Framework Membranes for Tunable Gas Separations. *ACS Appl. Mater. Interfaces* **2018**, *10*, 5586-5593.

[S95] He, G.; Dakhchoune, M.; Zhao, J.; Huang, S.; Agrawal, K. V. Electrophoretic Nuclei Assembly for Crystallization of High-Performance Membranes on Unmodified Supports. *Adv. Funct. Mater.* **2018**, *28*, 1707427.

[S96] Hou, J.; Sutrisna, P. D.; Zhang, Y.; Chen, V. Formation of Ultrathin, Continuous Metal–Organic Framework Membranes on Flexible Polymer Substrates. *Angew. Chem., Int. Ed.* **2016**, *55*, 3947-3951.

[S97] Shamsaei, E.; Lin, X.; Low, Z.-X.; Abbasi, Z.; Hu, Y.; Liu, J. Z.; Wang, H. Aqueous Phase Synthesis of ZIF-8 Membrane with Controllable Location on an Asymmetrically Porous Polymer Substrate. *ACS Appl. Mater. Interfaces* **2016**, *8*, 6236-6244.

[S98] Ban, Y.; Li, Z.; Li, Y.; Peng, Y.; Jin, H.; Jiao, W.; Guo, A.; Wang, P.; Yang, Q.; Zhong, C.; Yang, W. Confinement of Ionic Liquids in Nanocages: Tailoring the Molecular Sieving Properties of ZIF-8 for Membrane-Based CO_2 Capture. *Angew. Chem., Int. Ed.* **2015**, *54*, 15483-15487.

[S99] Huang, A.; Caro, J. Covalent Post-Functionalization of Zeolitic Imidazolate Framework ZIF-90 Membrane for Enhanced Hydrogen Selectivity. *Angew. Chem., Int. Ed.* **2011**, *50*, 4979-4982.

[S100] Huang, A.; Liu, Q.; Wang, N.; Caro, J. Organosilica functionalized zeolitic imidazolate framework ZIF-90 membrane for CO_2/CH_4 separation. *Micropor. Mesopor. Mater.* **2014**, *192*, 18-22.

[S101] Cacho-Bailo, F.; Etxeberría-Benavides, M.; Karvan, O.; Téllez, C.; Coronas, J. Sequential amine functionalization inducing structural transition in an aldehyde-containing zeolitic imidazolate framework: application to gas separation membranes. *CrystEngComm* **2017**, *19*, 1545-1554.

[S102] Cacho-Bailo, F.; Seoane, B.; Téllez, C.; Coronas, J. ZIF-8 continuous membrane on porous polysulfone for hydrogen separation. *J. Membr. Sci.* **2014**, *464*, 119-126.

[S103] Bux, H.; Liang, F.; Li, Y.; Cravillon, J.; Wiebcke, M.; Caro, J. Zeolitic Imidazolate Framework Membrane with Molecular Sieving Properties by Microwave-Assisted Solvothermal Synthesis. *J. Am. Chem. Soc.* **2009**, *131*, 16000-16001.

[S104] Liu, Q.; Wang, N.; Caro, J.; Huang, A. Bio-Inspired Polydopamine: A Versatile and Powerful Platform for Covalent Synthesis of Molecular Sieve Membranes. *J. Am. Chem. Soc.* **2013**, *135*, 17679-17682.

[S105] Pan, Y.; Wang, B.; Lai, Z. Synthesis of ceramic hollow fiber supported zeolitic imidazolate framework-8 (ZIF-8) membranes with high hydrogen permeability. *J. Membr. Sci.* **2012**, *421-422*, 292-298.

[S106] Zhang, X.; Liu, Y.; Li, S.; Kong, L.; Liu, H.; Li, Y.; Han, W.; Yeung, K. L.; Zhu, W.; Yang, W.; Qiu, J. New Membrane Architecture with High Performance: ZIF-8 Membrane

Supported on Vertically Aligned ZnO Nanorods for Gas Permeation and Separation. *Chem. Mater.* **2014**, *26*, 1975-1981.

[S107] Liu, Y.; Wang, N.; Pan, J. H.; Steinbach, F.; Caro, J. In Situ Synthesis of MOF Membranes on ZnAl-CO_3 LDH Buffer Layer-Modified Substrates. *J. Am. Chem. Soc.* **2014**, *136*, 14353-14356.

[S108] Huang, A.; Wang, N.; Kong, C.; Caro, J. Organosilica-Functionalized Zeolitic Imidazolate Framework ZIF-90 Membrane with High Gas-Separation Performance. *Angew. Chem., Int. Ed.* **2012**, *51*, 10551-10555.

[S109] Eum, K.; Hayashi, M.; De Mello, M. D.; Xue, F.; Kwon, H. T.; Tsapatsis, M. ZIF-8 Membrane Separation Performance Tuning by Vapor Phase Ligand Treatment. *Angew. Chem., Int. Ed.* **2019**, *131*, 16542-16546.

[S110] Wang, Y.; Jin, H.; Ma, Q.; Mo, K.; Mao, H.; Feldhoff, A.; Cao, X.; Li, Y.; Pan, F.; Jiang, Z. A MOF Glass Membrane for Gas Separation. *Angew. Chem., Int. Ed.* **2020**, *59*, 4365-4369.

[S111] Nian, P.; Li, Y.; Zhang, X.; Cao, Y.; Liu, H.; Zhang, X. ZnO Nanorod-Induced Heteroepitaxial Growth of SOD Type Co-Based Zeolitic Imidazolate Framework Membranes for H_2 Separation. *ACS Appl. Mater. Interfaces* **2018**, *10*, 4151-4160.

[S112] Kang, Z.; Xue, M.; Fan, L.; Huang, L.; Guo, L.; Wei, G.; Chen, B.; Qiu, S. Highly selective sieving of small gas molecules by using an ultra-microporous metal–organic framework membrane. *Energy Environ. Sci.* **2014**, *7*, 4053-4060.

[S113] Dou, H.; Xu, M.; Jiang, B.; Wen, G.; Zhao, L.; Wang, B.; Yu, A.; Bai, Z.; Sun, Y.; Zhang, L.; Chen, Z.; Jiang, Z. Bioinspired Graphene Oxide Membranes with Dual Transport Mechanisms for Precise Molecular Separation. *Adv. Funct. Mater.* **2019**, *29*, 1905229.

[S114] Zhao, J.; He, G.; Huang, S.; Villalobos, L. F.; Dakhchoune, M.; Bassas, H.; Agrawal, K. V. Etching gas-sieving nanopores in single-layer graphene with an angstrom precision for high-performance gas mixture separation. *Sci. Adv.* **2019**, *5*, eaav1851.

[S115] Yang, J.; Gong, D.; Li, G.; Zeng, G.; Wang, Q.; Zhang, Y.; Liu, G.; Wu, P.; Vovk, E.; Peng, Z.; Zhou, X.; Yang, Y.; Liu, Z.; Sun, Y. Self-Assembly of Thiourea-Crosslinked Graphene Oxide Framework Membranes toward Separation of Small Molecules. *Adv. Mater.* **2018**, *30*, 1705775.

[S116] Huang, S.; Dakhchoune, M.; Luo, W.; Oveisi, E.; He, G.; Rezaei, M.; Zhao, J.; Alexander, D. T. L.; Züttel, A.; Strano, M. S.; Agrawal, K. V. Single-layer graphene membranes by crack-free transfer for gas mixture separation. *Nat. Commun.* **2018**, *9*, 2632.

[S117] Huang, S.; Li, S.; Villalobos, L. F.; Dakhchoune, M.; Micari, M.; Babu, D. J.; Vahdat, M. T.; Mensi, M.; Oveisi, E.; Agrawal, K. V. Millisecond lattice gasification for high-density CO_2- and O_2-sieving nanopores in single-layer graphene. *Sci. Adv.* **2021**, *7*, eabf0116.

[S118] Yuan, Z.; He, G.; Faucher, S.; Kuehne, M.; Li, S. X.; Blankschtein, D.; Strano, M. S. Direct Chemical Vapor Deposition Synthesis of Porous Single-Layer Graphene Membranes with High Gas Permeances and Selectivities. *Adv. Mater.* **2021**, *33*, 2104308.

[S119] Schlichting, K.-P.; Poulikakos, D. Selective Etching of Graphene Membrane Nanopores: From Molecular Sieving to Extreme Permeance. *ACS Appl. Mater. Interfaces* **2020**, *12*, 36468-36477.

[S120] Song, Q.; Jiang, S.; Hasell, T.; Liu, M.; Sun, S.; Cheetham, A. K.; Sivaniah, E.; Cooper, A. I. Porous Organic Cage Thin Films and Molecular-Sieving Membranes. *Adv. Mater.* **2016**, *28*, 2629-2637.

[S121] Lu, H.; Wang, C.; Chen, J.; Ge, R.; Leng, W.; Dong, B.; Huang, J.; Gao, Y. A novel 3D covalent organic framework membrane grown on a porous α-Al_2O_3 substrate under solvothermal conditions. *Chem. Commun.* **2015**, *51*, 15562-15565.
